# VISproPT: A high-precision instrument for 3D shape analysis of parabolic trough panels

**DOI:** 10.12688/openreseurope.15967.1

**Published:** 2023-07-10

**Authors:** Marco Montecchi, Giuseppe Cara, Arcangelo Benedetti

**Affiliations:** 1 Solar Thermal, Thermodynamic and Smart Network Division, ENEA, ENEA-Casaccia via Anguillarese 301, Rome, 00123, Italy

**Keywords:** Concentrating Solar Power, parabolic-trough, reflective panels, 3D shape, optical profilometer

## Abstract

In Concentrating Solar Power plants, reflective panels are used to redirect solar radiation towards a receiver. Because the panel shape drives the radiation distribution around the focus where the receiver is placed, the 3D measurement is fundamental to assess the panel shape quality. The VISproPT instrument is the advanced version of the prototype VISprofile; these instruments are designed for indoor measuring of the 3D shape of parabolic-trough reflective panels. The VIS proPT hardware has been manufactured by MARPOSS Italia Spa and funded by EU project ’Solar Facilities for the European Research Area - Third Phase’ (SFERA-III), while the image processing software as well as the calibration procedure, based on the measurement of a perfectly flat surface as that of a calm body of water, have been developed by Italian National Agency for New Technologies, Energy and Sustainable Economic Development (ENEA). The instrument precision is better than 0.1 mrad and 0.3 mm (root mean square value over an area 1.2 × 0.8 m ^2^), for slope and height of the surface respectively. Technical details of experimental set up and calibration-procedure are here reported. The instrument effectiveness is shown by reporting the results obtained on a set of 10 specimen (5 inner + 5 outer) adopted to run the SFERA-III WP10 Task3 round-robin on 3D shape measurements among the different instruments used by the Fraunhofer Institute for Solar Energy Systems ISE (F-ISE), Deutsches Zentrum Fuer Luft - und Raumfahrt EV (DLR), the National Renewable Energy Laboratory (NREL) and Sandia National Laboratories (SANDIA).

## Plain language summary

The VISproPT is a novel instrument useful for scientists and technicians dealing with reflective panels for parabolic-trough (PT) Concentrating Solar Power (CSP) plants. The VISproPT is an advanced laboratory instrument with high precision designed to verify the shape compliance of PT panels. Deviations of slope and height from the ideal parabolic profile are evaluated and presented in terms of 2D contour maps as well as root mean square (RMS) values; these geometrical parameters are very useful to improve the production technology. Concerning the panel effectiveness in redirecting the solar radiation onto the linear receiver tube, the most representative parameter is the so-called *intercept-factor* (I-F), i.e. the ratio of the geometrical intersection of the reflected solar beam with the receiver tube; the VISproPT software evaluates I-F by considering the standard divergence of the solar radiation on the Earth (4.7 mrad, half-apex angle), and the proper values of parabola focal-length and receiver-tube diameter; I-F is reported as a 2D contour map and its mean value.

## 1 Introduction

The Visual Inspection System (VIS) approach was patented by ENEA on 2008 ^1^; it is based on the idea of placing a light source nearby the focus of the reflector and acquiring a number of images in the near-field from different positions of camera or source. On the basis of the VIS approach we developed the following instruments:

1. VISfield to verify the mutual optical alignment between receiver tube and parabolic trough reflector for modules in field ^2^
2. VISshed, the adaptation of VISfield for the quality control in the shed, soon after the module assembling ^3^
3. VISdish for facet-canting and 3D shape measurement of solar dish in field ^4^
4. VISproLF for 3D shape measurements of Linear Fresnel panels in laboratory/industry ^5^
5. VISprofile for 3D shape measurement of parabolic trough panels in laboratory

The last one was developed in 2009 at ENEA Casaccia just as demonstrative experimental set-up, alternative to the older 3D optical profilometer ^6^; the VISprofile was scarcely engineered and unsuitable to industry. On the other hand, shape/slope deviation of the reflective surface from the ideal profile causes solar radiation leakage with reduction of efficiency. That makes the shape quality check a very important task. Therefore, under ENEA guidance, since 2012 the Italian worldwide leader in Measurement, Inspection and Testing, MARPOSS ^7^ started to engineer a new version, named VISproPT, suitable to commercial purposes. The prototype was soon ready but because of weakness of CSP market and insufficient ENEA funding, it remained in stock until 2018 when it was delivered to ENEA Casaccia to be acquired under the umbrella of EU SFERA-III project ^8^. The hardware commissioning was delayed to September 2021 because of the COVID-19 pandemic and the resulting shortage of electronic components. Only from that time the development of image processing software as well as calibration procedure started. The good side of such a long gestation is the relevant progress, matured in the meanwhile, of the ENEA knowhow on the use of digital cameras in 3D geometric characterization; as a result now the instrument calibration is much simpler than what initially prefigured. To exploit the new instrument we took the opportunity of WP10 Task3 of SFERA-III project ^8^ to launch the proposal of a new round-robin (RR) on 3D shape measurements of panels for parabolic-trough (PT) solar collectors; a previous attempt, accomplished about 10 year ago in SolarPACES TaskIII framework ^9^, did not give satisfactory results because of the difference among the results obtained by the participants were greater than the experimental error; as matter of a fact, today a dedicated guideline on the topic is still missing. The proposal was accepted by F-ISE and DLR and then has been extended to National Renewable Energy Laboratory (NREL) and Sandia National Laboratories (SANDIA) who benefit of the Transnational Access tools offered by SFERA-III to send teams in Europe for visiting research infrastructures and participating to the introductory meeting on the round robin itself.

At the time of writing this article, the round-robin is just started; the global results will be published in a next future. This paper is focused on the novel instrument VISproPT; the results achieved by ENEA are here presented just to illustrate the instrument functionality.

## 2 VISproPT basis

### 2.1 Main hardware components

In general, the VIS method ^1^ just needs a good monochrome digital camera and a structured light source; the source type and the arrangement of these components around the object to be measured depend on the specific case.

The engineered version of the VISprofile is named VISproPT to distinguish it from the other instrument, named VISproLF ^5^, designed for 3D shape measurements of panels for linear Fresnel collectors.

**Figure 1.**
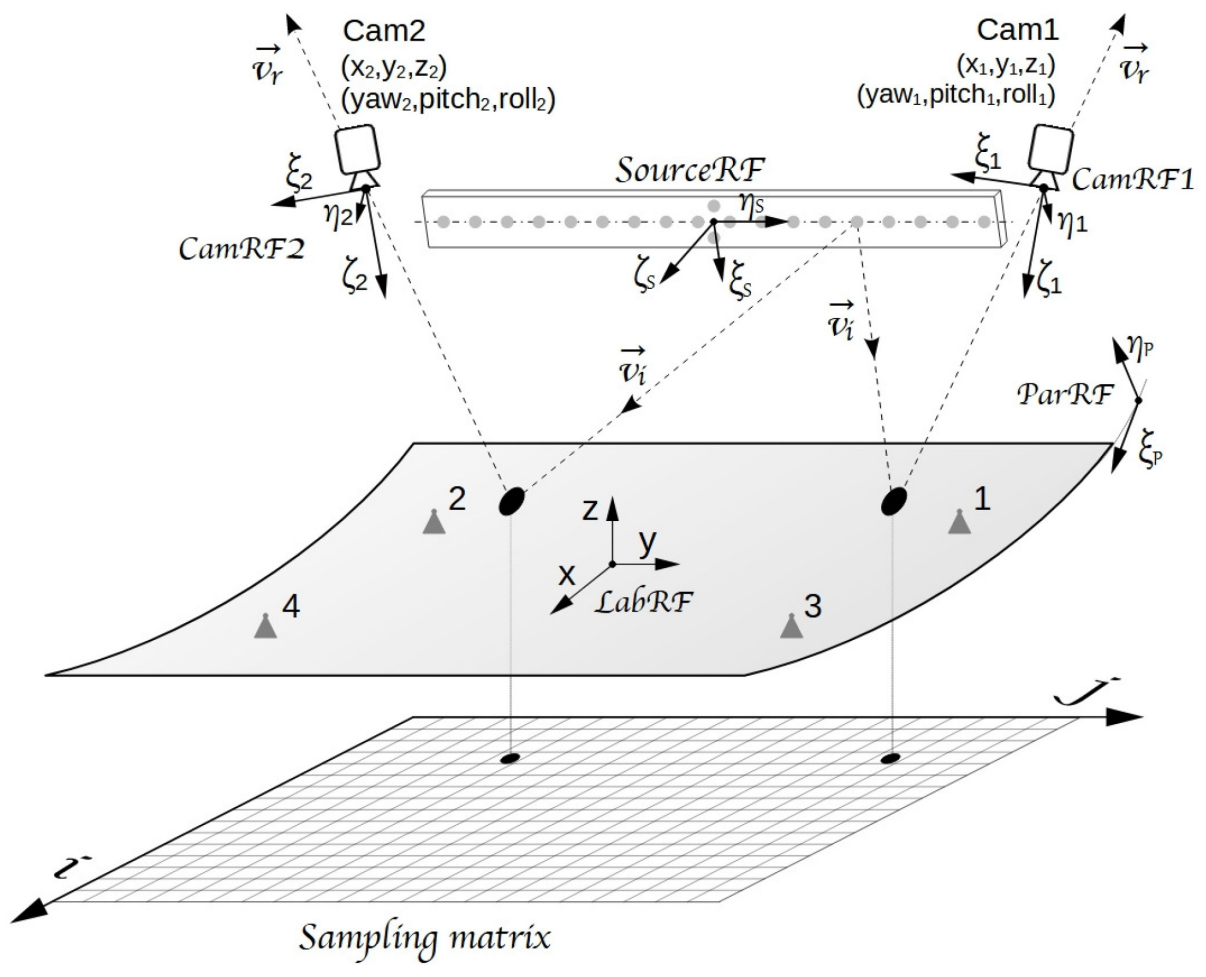
VISproPT optical sketch: two digital cameras (Cam1 and Cam2) acquire photos of the panel surface with the reflected images of the point source array. Five reference frames are used in the image-processing: 1) parabola ( *ParRF*), 2) laboratory ( *LabRF*), 3) point source array ( *SourceRF*), 4) Cam1 ( *CamRF1*), 5) Cam2 ( *CamRF2*). At the end of the image processing, the experimental values of height and partial derivatives of the surface are gridded over the *sampling matrix*.

**Figure 2.**
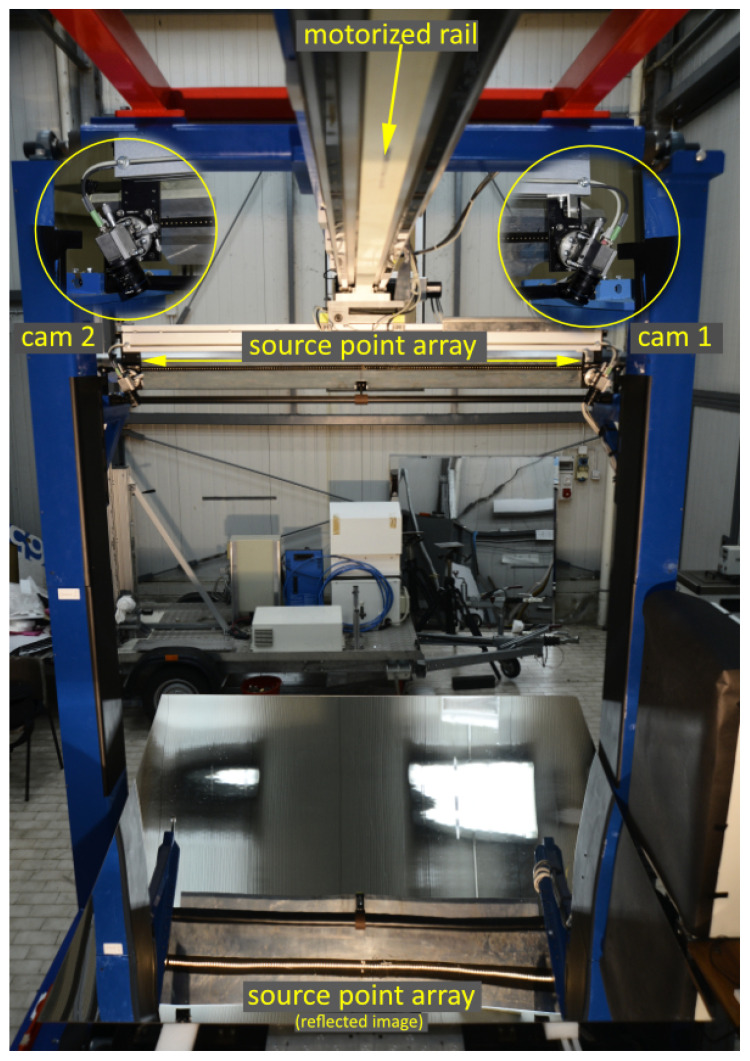
VISproPT hardware. The main components are: the point source array, two digital cameras and a motorized rail for moving the camera over the specimen.

The optical sketch adopted in VISproPT is shown in Figure 1, while the hardware, manufactured by MARPOSS Italia Spa ^7^, in Figure 2. The investigated PT panel is placed on 4 supports (1,2,3,4) aligned on the same horizontal plane and positioned accordingly to the panel design. The origin of the laboratory reference frame LabRF is about in the center of the 4 attaching points, with the Z axis aligned along the vertical and the X axis parallel to the motorized rail (and the direction of curvature of the panel).

The motorized rail is horizontal at about *z* = 1.5 m; its alignment was optimized by a Total Station Leica TDA 5005 ^10^. The rail is long enough (about 3 m) to obtain that the point source images, reflected by the panel and viewed by the cameras, scan the entire panel surface during the linear translation of the cameras along a suitable range.

The light source is composed by a off-the-shelf low-cost LED strip wrapped in a metallic box where one face, made of an aluminum thick plate, has been precisely drilled by means of a computer numerical control machine; that makes our linear array of point sources with step 10.00 mm. Thanks to the chosen materials and the specific design, the array of point sources can be assumed as perfectly linear with accuracy better than 0.01 mm for each point, in anyone of the three dimensions.

The central point of the array is marked by a couple of point sources symmetrically displaced (10.00 mm) from the array central-line; as will be explained later, that detail is fundamental to allow the proper attribution of the origin point source for each one of the reflected-point images displayed in the images captured by the cameras.

By means of the Total Station the point source array is aligned parallel to the Y axis, with its center in (−0.8 , 0 , 1.5) m. In that condition only the Pitch angle is not null; it was adjusted to about -30.0 deg.

Concerning to the digital camera, the instrument makes use of two identical units composed by:

- camera: Baumer VCXG-51M, 2448 px x 2048 px complementary metal-oxide semiconductor (CMOS) sensor, monochrome
- objective: Opto Engineering EN5MP0816 5 Megapixel 8 mm 1:1.6 2/3”

The two cameras are fastened on a road attached to the motorized rail; the road is horizontal and parallel to the Y axis. The adoption of two cameras allows to keep constant the width of the scanned area along their linear translation; to that purpose each camera is positioned at about the same y-coordinate of the underlying curved-rims of the PT panel; the cameras are oriented in such a way to frame the entire width of the panel (in the flat direction) with the widest side of the CMOS sensor. Thanks to the procedure described in Section 2.5, the two cameras can be positioned and oriented without paying particular attention.

At author’s knowledge the VISproPT instrument is totally original for both hardware configuration (the optical sketch) and the approach adopted to evaluate the 3D shape of PT panels.

### 2.2 Software

The hardware provided by MARPOSS includes an industrial computer hosting a software for the basic instrument management, with commands:

- go to home position
- go to *x*
- acquire a single image (one for each camera) at the given position
- scan the panel surface from *x _max_* to *x _min_*, acquiring images with step *x _step_*

This MARPOSS software is not provided as part of this article because proprietary; on the other hand, it is strictly related to the hardware components adopted in the VISproPT, and not useful for others. In other words, if the reader wanted to develop a similar instrument, he would have to write his own software equipped with the libraries necessary for managing the chosen hardware components (image acquisition by the cameras and motorized-rail handling). Thus the omission of the MARPOSS software does not limit the readers from replicating the methods and data processing described in this article.

The development of the software for instrument-calibration and image-processing has been totally demanded to ENEA which holds the full know-how of the VIS method. For the sake of simplicity this software is hosted on the Lab PC from which one can pilot the instrument by sending command-scripts to the industrial computer. As drawback, the sequence of acquired images must be transferred to the Lab PC before they can be processed; on the other hand, in future, the new software could be directly installed in the industrial computer.

The main features of the software developed by ENEA are:

1. C++ written;
2. provided with a Qt graphical user interface ^11^;
3. based on the OpenCV library ^12^ which includes a rich tool-set for computer vision;
4. equipped with nonlinear least squares offered by CMINPACK library ^13^.

This software now covers all the steps of 3D shape measurements:

- Camera-lens calibration, for image undistortion
- Instrument calibration
- Image-processing for evaluating: i) 3D shape (slopes d *z*/d *x*, d *z*/d *y* and height *z*), ii) deviations from the ideal shape and, last but not least, iii) evaluation of the intercept factor at a given longitudinal angle.

To add value to the work done so far, believing in knowledge sharing, we took the opportunity offered by Open Research Europe to make the VISproPT software available for everyone as open source software under the GNU General Public License as published by the Free Software Foundation version 3. Code source files and data of one exemplary measurement can be freely downloaded from *Software availability* and *Data availability* ^14, 15^.

Although already described in 5, in order to make fully understandable the image processing we developed for VISproPT, the next three paragraphs shortly recall some well assessed arguments, such as: pinhole camera model, camera calibration, transformation rules between two reference frames, and determination of position and attitude of the camera.

### 2.3 Pinhole camera model and camera calibration

**Figure 3.**
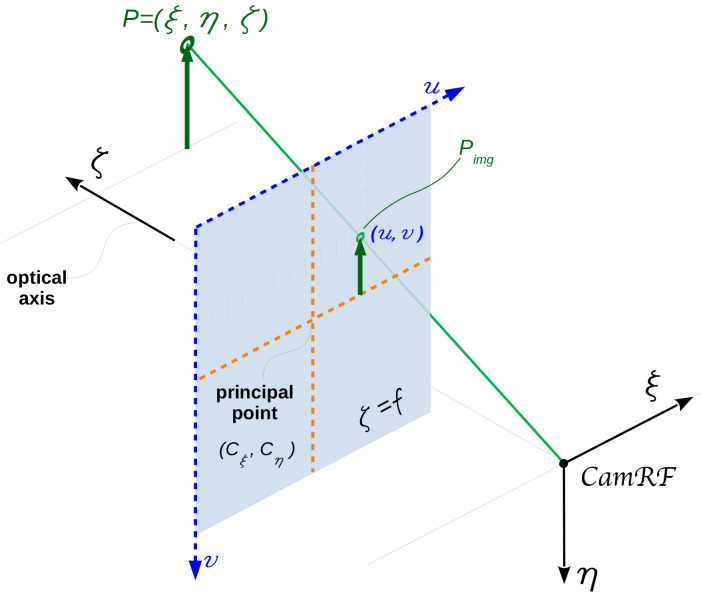
Pinhole camera model. Source point *P*, image point *P _img_*, and lens center (the origin of the camera reference frame *CamRF*) are aligned along the same straight line.

The *pinhole camera model* ^12^, shown in Figure 3, is commonly used for 3D reconstruction from 2D images. Let us consider the camera reference frame (CamRF) depicted in the figure: the 3D point *P* = ( *ξ*, *η*, *ζ*) is imaged in the point *P _img_* = ( *u*, *v*) on the sensor (commonly, CCD or CMOS) which is set on the plane *ζ* = *f* orthogonal to the optical axis, i.e. the *ζ*-axis; the origin of CamRF is the *lens center*; the *ζ*-axis crosses the sensor at the *principal point* with pixel coordinates ( *c _ξ_*, *c _η_*); the sensor is unrealistically set between lens-center and object to take into account of the image straightening provided by the sensor electronic; *ξ*-axis ( *η*-axis) is parallel to sensor rows (columns). In the pinhole camera model, source ( *P*), image ( *P _img_*) and lens-center (the origin of CamRF) are on the same straight line, thus

$$u=f_{\xi }\frac{\xi }{\zeta }+c_{\xi }\;(1)$$

$$v=f_{\eta }\frac{\eta }{\zeta }+c_{\eta }\;(2)$$

where *f _ξ_* and *f _η_* are the focal lengths, and ( *c _ξ_*, *c _η_*) are the coordinates of the principal point; all these parameters are expressed in pixel units and stored in the so-called *camera matrix*. If the pixel is a perfect square, like for the here adopted cameras, *f _ξ_* = *f _η_* = *f*. It must be noted that the principal point is close to the sensor center, but generally it is not coincident with it.

Unfortunately any real lens usually induce some *distortions* (mostly radial) in the image. In order to make the pinhole camera model valid, one has first to *undistort* the image on the basis of *camera matrix* and *distortion coefficients*; those are obtained by calibrating the *camera-lens* system; please note that the evaluation of focal length *f* and principal point ( *c _ξ_*, *c _η_*) is part of that calibration. Noteworthy, camera matrix and distortion coefficients are strictly related to the specific camera-lens system, so that even if only the focus is slightly changed, one must repeat the whole calibration procedure. Procedure and functions for camera calibration and image correction are part of the rich OpenCV library ^12^ to which we refer the reader for further information. Here we just recall that calibration is obtained by processing a sequence of images of the same chessboard differently oriented. Such a chessboard has to be printed and stitched on a rigid flat substrate by the user. We used a black and white chessboard composed by 54 × 46 squares with 10.0 mm side.

### 2.4 Transformation rules

As shown in Figure 1, five reference-frames play an important role in VISproPT:

- the first two, named CamRF1 and CamRF2, are related to the cameras (as detailed in Figure 3)
- the third is the Laboratory frame (LabRF), which origin is set in the center of the four attaching points, with the *z*-axis aligned along the vertical, and the *x*-axis parallel to the motorized rail
- the fourth reference-frame, named SourceRF, is related to the point source array: it is centered between the two special central points, with the y-axis along the array, and z-axis orthogonal to the drilled-face of the metallic box
- the fifth (ParRF) is the natural reference frame of the ideal parabola, where $\eta =\frac{1}{4f_{p}}\xi^{2}$ with *f _p_* parabola focal length

The 3D coordinates of a point in one of these reference-frames can be expressed in those of any other by translation and rotation. The translation rules are well known; conversely for rotations there are dozen of different conventions, depending on the considered angles and the sequence of rotations around the axes ^16^. Among them we chosen the one normally used for aircraft, where the association between angle and axes is: Yaw → *ζ*, Pitch → *η* and Roll → *ξ*. They are applied in that order.

The transformation of the coordinates ( *ξ*, *η*, *ζ*) from CamRF, SourceRF or ParRF (the plane) to LabRF ( *x*, *y*, *z*) (the Earth) are

$$\begin{array}{l} \;x=c_{1}c_{2}\xi +(c_{1}s_{2}s_{3}-c_{3}s_{1})\eta +(s_{1}s_{3}+c_{1}c_{3}s_{2})\zeta \\ \{\;y=c_{2}s_{1}\xi +(c_{1}c_{3}+s_{1}s_{2}s_{3})\eta +(c_{3}s_{1}s_{2}-c_{1}s_{3})\zeta \\ \;z=-s_{2}\xi +c_{2}s_{3}\eta +c_{2}c_{3}\zeta \end{array}\;(3)$$

where *s _j_* and *c _j_* are respectively sin and cos with argument *j* = 1 *→* Yaw, *j* = 2 *→* Pitch, *j* = 3 *→* Roll.

With the same symbolism, the opposite transformation from Earth (LabRF) to plane (CamRF, SourceRF or ParRF) are

$$\begin{array}{l} \;\xi =c_{2}c_{3}x-c_{2}s_{3}y+s_{2}z\\ \{\;\eta =(c_{1}s_{3}+c_{3}s_{1}s_{2})x+(c_{1}c_{3}-s_{1}s_{2}s_{3})y-c_{2}s_{1}z\\ \;\zeta =(s_{1}s_{3}-c_{1}c_{3}s_{2})x+(c_{3}s_{1}-c_{1}s_{2}s_{3})y+c_{1}c_{2}z \end{array}\;(4)$$

Equation 3 and Equation 4 represents only the rotation; the translation is applied after the rotation.

### 2.5 Position and attitude of cameras

**Figure 4.**
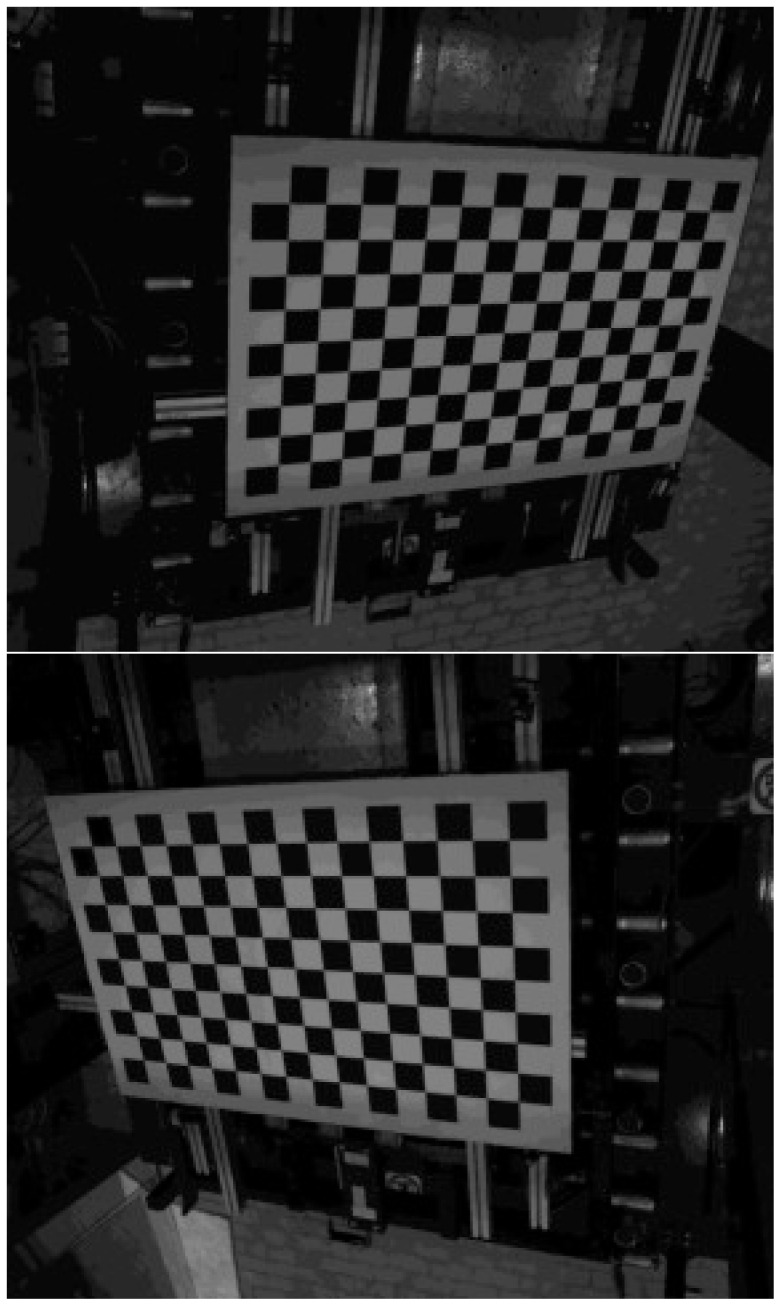
View of the chessboard 15 × 9 = 135 corners from Cam1 (left) and Cam2 (right).

Position and attitude of each camera in LabRF are fundamental parameters. They can be easily obtained by analyzing the image of a chessboard placed on the specimen holder (see Figure 4). The chessboard is precisely positioned by means of the Total Station.

Our chessboard consists of 16 × 10 squares, resulting in 15 × 9 corners, i.e. the points where the corners of 4 squares (2 white and 2 black) are touching one to each other; the step along columns and rows is the same, 70.0 mm.

Each one of the two images shown in Figure 4, is analyzed for evaluating the pixel-coordinates ( *u*, *v*) of each 15×9 = 135 corners in the proper CamRF. This job can be automatically accomplished by using the function findChessboardCorners of the OpenCV library ^12^.

On the other hand the coordinates of these corners can be easily computed in LabRF because the chessboard is: i) aligned on the horizontal plane defined by the *X Y* axes; ii) centered in the origin (0, 0, 0); iii) oriented with rows (columns) parallel to *Y* ( *X*) axis. Under these conditions the coordinate of the corner at row *i* = 1, 2, .., *N _i_* − 1 and column *j* = 1, 2, .., *N _j_* − 1 are

$$\begin{array}{l} \;x_{j,i}=(-\frac{N_{i}}{2}+i)\Delta \\ \{\;y_{j,i}=(-\frac{N_{j}}{2}+j)\Delta \\ \;z_{j,i}=0 \end{array}\;(5)$$

where Δ = 70.0 mm.

When corner-coordinates, lens-center-position ( *x _c_*, *y _c_*, *z _c_*) and camera-attitude (Yaw,Pitch,Roll) are known in LabRF, one can evaluate the expected position of each corner in the acquired image by means of the pinhole camera model: in LabRF the corner ( *j*, *i*) is located at the end of the vector

$${\vec{v}}_{j,i}=(x_{j,i}-x_{c},y_{j,i}-y_{c},z_{j,i}-z_{c})\;(6)$$

applied to the camera lens-center. By means of Equation 4 the components of ${\vec{v}}_{j,i}$ in LabRF (given in Equation 6) can be transformed in CamRF, obtaining ( *ξ*_*j*, *i*_, *η*_*j*, *i*_, *ζ*_*j*, *i*_). Then, according to the pinhole camera model, the image of the corner ( *i*, *j*) is expected in

$${\vec{u}}_{j,i}=\frac{f}{\zeta_{j,i}}(\xi_{j,i},\eta_{j,i},\zeta_{j,i})=(u_{j,i}-c_{\xi },v_{j,i}-c_{\eta },f)\;(7)$$

where ( *u*_*j*, *i*_, *v*_*j*, *i*_) are its pixel coordinate.

The comparison between experimental and expected values of the corner pixel-coordinates is a powerful method for optimizing the values of ( *x _c_*, *y _c_*, *z _c_*) and (Yaw,Pitch,Roll). More precisely we adopted the modified Levenberg Marquardt non linear least square algorithm, offered by CMINPAK library ^13^ on the differences between calculated and experimental corner pixel coordinates. The best fit procedure is launched starting from a rough (but realistic!) initial evaluation of position and attitude of the camera. The procedure is separately applied to each camera.

### 2.6 Instrument calibration

Unfortunately we realized that the evaluation of principal point ( *c _ξ_*, *c _η_*) and focal length *f* by the chessboard, explained in Section 2.3, is not precise enough for our purposes: we found that as the number of images considered for the camera calibration increases, the succession of estimated values does not seem to converge because at each step the result is affected by the portion of the visual field occupied by the chessboard in the new added image. This weak point has a cascade effect on the accuracy of the evaluation of lens-center position and camera-attitude described in Section 2.5. 

**Figure 5.**
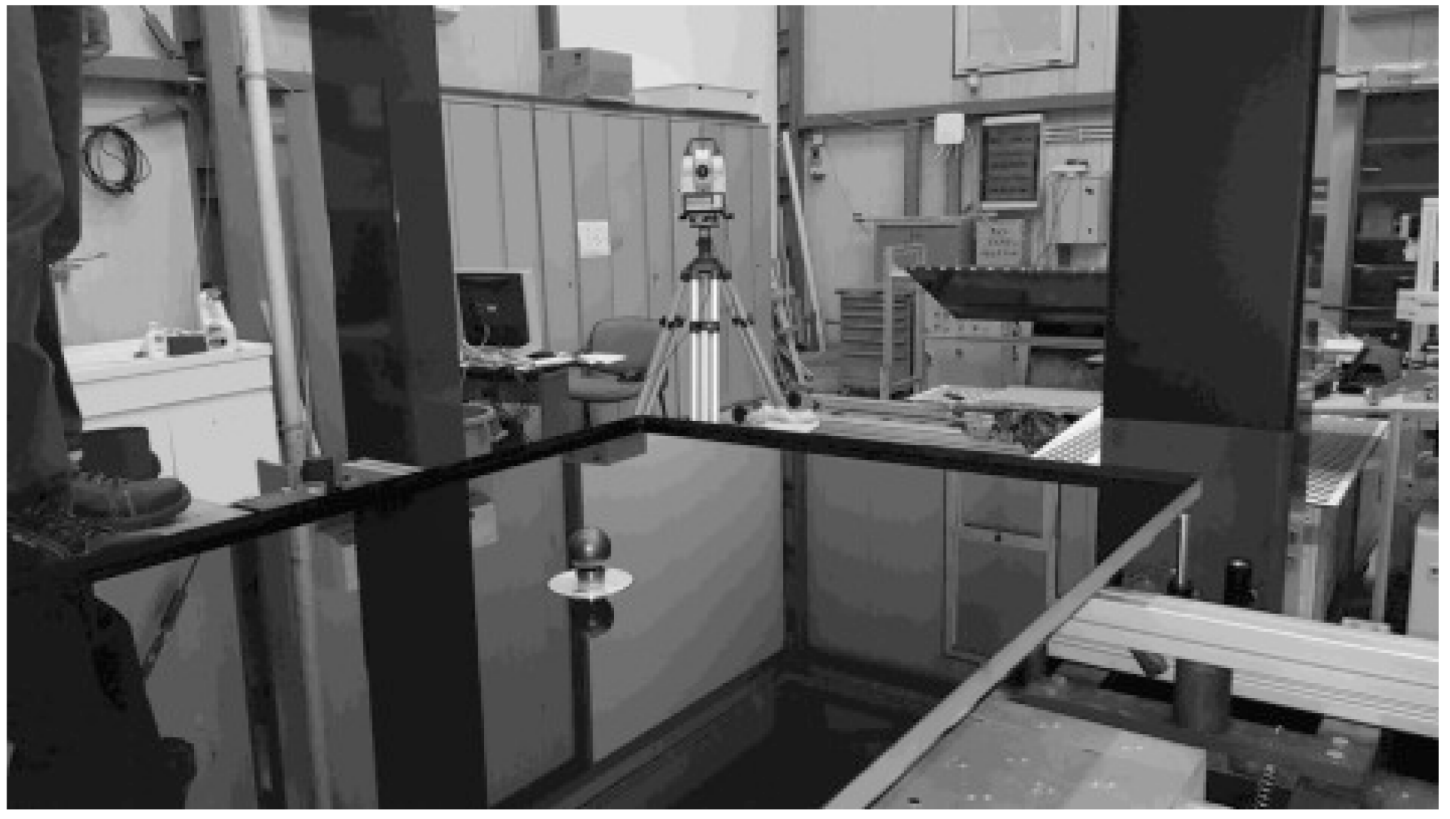
Basin with water and corner cube reflector for z measurement by Leica Total Station.

To overcome this problem we enriched the software with the final instrument-optimization: principal point, focal length, lens-center position and attitude of each camera are optimized with the modified Levenberg Marquardt non linear least square algorithm, by minimizing the slope deviations of the 3D shape measurement of a perfectly flat surface as that of a calm body of water. Thanks to the laboratory’s remoteness from busy streets, undergrounds and railways, once calm, the water surface remains totally unperturbed. As shown in Figure 5 a basin (1.2 × 0.8 m ^2^) is placed on the attaching points of the VISproPT; once again the z coordinate of the interface air|water was evaluated by means of the Total Station. Moreover, said *x _cj_* and *z _cj_* two of the three coordinates in LabRF of the lens center of the j-camera, the difference Δ *x*_21_ = *x*_*c*2_ − *x*_*c*1_ and Δ *z*_21_ = *z*_*c*2_ − *z*_*c*1_ can be directly measured by the Total Station and used as constrains in the best-fit by setting *x*_*c*2_ = *x*_*c*1_ + Δ *x*_21_ and *z*_*c*2_ = *z*_*c*1_ + Δ *z*_21_; thus *x*_*c*2_ and *z*_*c*2_ are derived parameters.

After the instrument calibration, the measured shape of the water surface results to be flat for better than 0.1 mrad and 0.3 mm (RMS values), respectively for slope and height of the surface with area 1.2 × 0.8 m ^2^, about.

### 2.7 Evaluation of the 3D shape

Once lens-center position, camera-attitude and point-source-array deployment are known in LabRF, the instrument is ready to perform 3D shape measurements. This section describes the original procedure we are proposing.

The measurement is based on the analysis of the two sequences of images acquired by the two cameras along their translation *x _max_* → *x _min_*; the range of the translation is properly set to maximize the scan area of the specimen surface, but taking care to avoid any loss in the sequence of the imaged point sources. As matter of fact, avoiding discontinuity between one point source and its neighborhoods is essential for the right association between imaged and origin point source, as will be clarified shortly.

**Figure 6.**
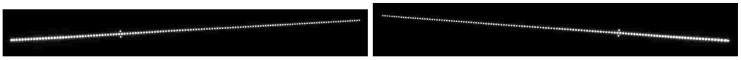
Point source array reflected by water and viewed by Cam1 (left) and Cam2 (right).

Differently to conventional deflectometry ^17^, VISproPT adopts a fully binary method. As an example Figure 6 shows the relevant part of a couple of images acquired for the instrument calibration. The images are monochrome with 8 bit pixel-depth, i.e. the luminosity ranges in [0, 255].

The automatic detection of the point sources contained in the image is fundamentally based on grey thresholding around a suitably set value (in this case 30): the luminosity of the pixels forming the point source image (technically named “blob”) is above the threshold, while all the pixels around have lower luminosity. Anyway additional constrains, as minimal blob area, minimal distance from two blobs, etc, can be set if necessary. To avoid false blob-detection, the laboratory must be sufficiently darkened.

Initially, the software performs two operations for any image of each sequence:

1. automatic detection of the blobs framed in the image by means of the SimpleBlobDetector Class offered by the OpenCV library (the luminosity threshold is one of the input parameters)
2. for each detected blob, the corresponding values of camera-number, frame-number, origin-point-source-number and ( *j*, *i*) pixel coordinates of the blob centroid are stored in the array

double results[550000]; /*
results[i*6+ 0] = N. frame
results[i*6+ 1] = N. Camera
results[i*6+ 2] = N. source
results[i*6+ 3] = /
results[i*6+ 4] = column blob-coordinate (px)
results[i*6+ 5] = row blob-coordinate (px) */


where i is the progressive number of the detected blob, and N.source is the index of the point source from which the blob originates; the software takes care of the association


blob↔N.source

starting from the recognition of the special doublet marking the center of the point source array (see Figure 6); from that, the proper point-source index is sequentially assigned to all the other surrounding blobs.

At the end of the images processing, the array results is populated with the data of all the blobs detected. Note that, unless one modify the distortion coefficients, the data stored in results do not depend on focal, central point, lens-center position and camera-attitude. This fact greatly reduces the computing time of the instrument calibration algorithm because the image-analysis has not to be repeated at each iteration of the best-fitting.

Then the second stage of the processing starts; it consists in the use of the data already stored in results to evaluate the parameters of the surface at each point of reflection; the new parameters are computed and stored in the multidimensional array


double S[Nj][Ni][12]={}; /*
S[j][i][0] = vn[0] \
S[j][i][1] = vn[1] - normal unit vector
S[j][i][2] = vn[2] /
S[j][i][3] = z_ideal (height) (mm)
S[j][i][4] = N. of independent evaluations
S[j][i][5] = dz/dx (partial y-derivative)
S[j][i][6] = dz/dy (partial x-derivative)
S[j][i][7] = z_exp (height) (mm)
S[j][i][8] = dz/dx_ideal
S[j][i][9] = dz/dy_ideal
S[j][i][10]= intFat
S[j][i][11]= z_exp rms */


where the indices [j][i] give the cell position (j-column and i-row) of the sampling matrix drawn in Figure 1, while the third index is used to store the several parameters associated to the cell.

More precisely the data-set of each blob stored in results is processed as follow:

- In CamRF, computing of the reflected unit vector $\vec{r}$ by the knowledge of the pixel coordinate of the blob ( *u*, *v*) and the central point ( *c _ξ_*, *c _η_*) of the system camera-lens (see Equation 7)
- Transformation of $\vec{r}$ in LabRF by Equation 3 using the knowledge of the lens-center coordinates and camera-attitude
- Calculation of the intersection between the straight line driven by $\vec{r}$ applied to the lens-center (with *x _c_* actualized to the proper frame-number) and the specimen surface represented by the *z*-data stored in S[j][i][7]; the knowledge of the reflection-point position allows one to determine the coordinate [j][i] of the cell containing it
- Calculation of the incidence unit vector $\vec{i}$ in LabRF by the knowledge of the position of the origin point source and the one of the point of reflection (as determined in the previous step)
- Calculation of the unit vector normal to the surface $\vec{n}$ ∝ –( $\vec{i}$ – $\vec{r}$ ) and, consequently, the partial derivatives along X and Y axis

We use to set the size of *S* matrix cell to the step-value used for the image acquisition during the camera translation, typically 10.0 mm. Generally each cell is filled with more than one point; the mean value of the normal unit vector is finally stored. Then the sub-region of *S* matrix really populated by experimental data is delimited and processed with an inpainting routine to fill the few cells not containing data.

Finally, 2D integration is accomplished starting from each one of the attaching point (where *z* is known); then the mean value is stored in S[j][i][7] while the RMS value in S[j][i][11].

At the first run the panel-shape is assumed to be the ideal one, that is S[j][i][7] = S[j][i][3]; then the whole procedure is repeated by using each time the refreshed value of S[j][i][7] obtained by the 2D integration; when the maximum difference between one iteration and the following is less than a threshold value (as an example 0.03 mm) the loop ends. Generally the convergence is reached after few tens of iterations.

Once the 3D shape of the panel is known, the intercept factor can be computed; the software consider the standard solar divergence of 4.7 mrad (half apex-angle) and the longitudinal incidence angle *θ _L_* set by the user.

As final step the software builds and saves as JPEG images the 2D contour maps representing the deviation of d *z*/d *x*, d *z*/d *y*, height *z* as well as the one of the intercept factor; here for *deviation* we refer to the difference between experimental and ideal values.

It is noteworthy that the above procedure is not restricted to parabolic trough panels: as a matter of fact it was successfully used to measure the water surface. Anyway the movement limited to the cameras, with the source in steady position, greatly limits the maximum size of the measurable area of flat specimens. On the other hand, the VISproPT hardware could be easily implemented by adding a linear translator to move the specimen along the *x*-axis, and scan the specimen with cameras and point source array in steady position. This alternative configuration could be useful for linear-Fresnel panels.

### 2.8 Ideal parabola reference frame

For evaluating the compliance of height *z* and slopes, d *z*/d *x* and d *z*/d *y*, observed in LabRF with the values expected for the ideal parabola, the knowledge of the relationships between LabRF and ParRF (where the profile is described by the canonical equation $\eta =\frac{1}{4f_{p}}\xi^{2}$ ) is fundamental.

**Figure 7.**
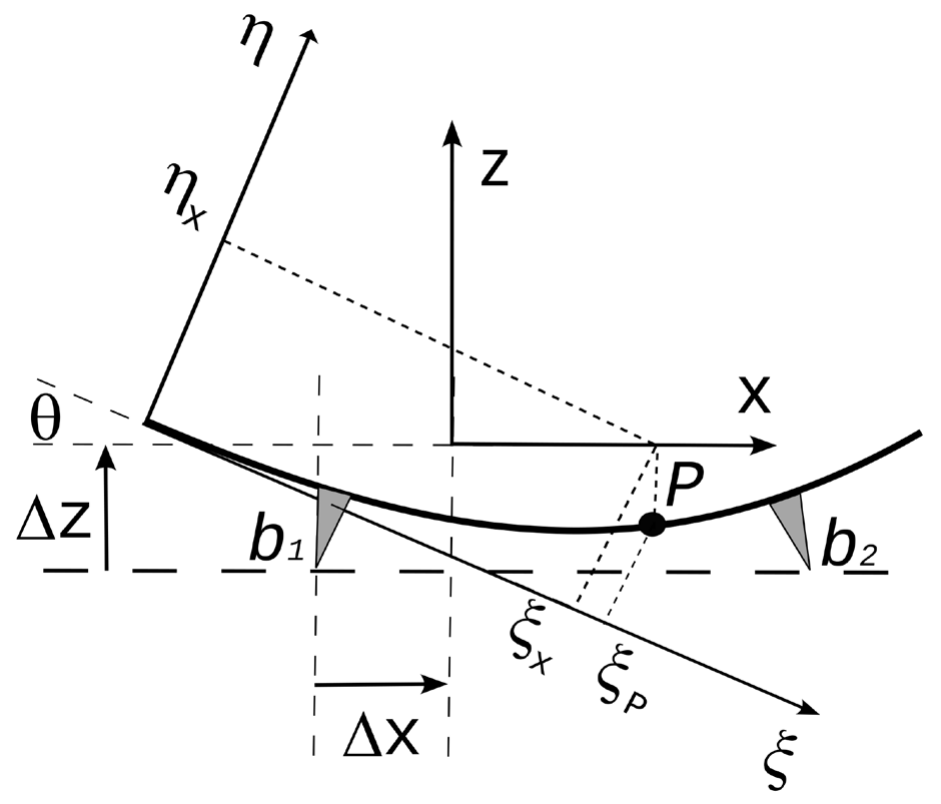
*ParRF* (parabola reference frame) and *LabRF* (lab reference frame); the center of the four supports (see Figure 1) lie over an horizontal plane parallel to the *X Y* plane of *LabRF*.

Referring to Figure 7, the angle *θ* between the *x* axis of LabRF and *ξ* axis of ParRF is given by

$$\theta =\arctan\left(\frac{\eta_{b_{2}}-\eta_{b_{1}}}{\xi_{b_{2}}-\xi_{b_{1}}}\right)\;(8)$$

where ( *ξ*_*b*_1__, *η*_*b*_1__) and ( *ξ*_*b*_2__, *η*_*b*_2__) are the coordinates in ParRF of the inner ( *b*_1_) and outer ( *b*_2_) supporting points.

Let Δ *x* and Δ *z* the signed increments to reach the LabRF origin from the center of *b*_1_; in ParRF the origin of LabRF is at

$$\begin{array}{l} \xi_{L}=\xi_{b_{1}}+\Delta x\;\cos\theta -\Delta z\;\sin\theta \\ \eta_{L}=\eta_{b_{1}}+\Delta x\;\sin\theta +\Delta z\;\cos\theta . \end{array}\;(9)$$

Let ( *x*, *y*, *z*) the coordinate of the measured point *P* of the panel surface. In order to compare *z* with the value expected for the ideal parabola, one needs to know the value of *ξ _P_*, i.e. the abscissa of *P* in ParRF. Developing the reasoning in the plane *y* = 0, the point ( *x*, 0, 0) in ParRF has coordinates

$$\begin{array}{l} \xi_{x}=\xi_{L}+x\;\cos\theta \\ \eta_{x}=\eta_{L}+x\;\sin\theta . \end{array}\;(10)$$

The coordinate of *P* ( *ξ _P_*, *η _P_*) are the solution of the equation system

$$\begin{array}{l} \eta =\frac{1}{4f_{p}}\xi^{2}\\ \eta -\eta_{x}=-\frac{1}{\tan\theta }(\xi -\xi_{x}) \end{array}\;(11)$$

having solution

$$\xi_{P}=\frac{-b+\sqrt{b^{2}-4ac}}{2a}\;(12)$$

where $a=\frac{1}{4f_{p}}$ $b=\frac{1}{\tan\theta }$ $c=-\eta_{x}-\frac{\xi_{x}}{\tan\theta }$ .

Finally, in LabRF

$$\begin{array}{l} x_{P}=(\xi_{P}-\xi_{L})\cos\theta +(\eta_{P}-\eta_{L})\sin\theta =x\\ z_{P}=-(\xi_{P}-\xi_{L})\sin\theta +(\eta_{P}-\eta_{L})\cos\theta \end{array}\;(13)$$

where $\eta_{P}=\frac{1}{4f_{p}}\xi_{P}^{2}$ .

Concerning the ideal slopes at point *P* in LabRF

$$\text{d}z/\text{d}x=\tan(\arctan(0.5*\xi_{P}/f)\;-\;\theta )\;(14)$$

dz/dy=0.(15)

Finally, the compliance of the measured 3D shape with the ideal one is given by the *deviation*, i.e. the difference between experimental and ideal values in LabRF already discussed in the end of Section 2.7.

## 3 Validation experiment

VISproPT was designed for measuring Italian style PT panels; they are characterized by the use of 1 mm thick solar mirrors glued to rigid parabolic-shaped sheet moulding compound substrates, 1200 mm wide; so that a PT module (12 m long) is composed by 40 panels, 20 inner and 20 outer; the focal length is 1810 mm.

Conversely the panels adopted in RR are for collector type LS3 with focal length 1710 ± 1 mm. The panel width is 1700±1 mm while the chord is 1624 and 1501 mm for inner and outer type, respectively; that width value is a bit over the limit of the actual VISproPT hardware: although the two cameras can be spaced 1.7 m apart (each one flying over one of the two curved-rims), the source array is not sufficiently long to be viewed reflected over more than half-width of the panel surface when the cameras are close to *x _max_*; therefore a small triangular area with base centered in the middle of the farthest rim from the vertex is not sampled; in the 2D contour maps that not-sampled area is gray painted. VISproPT was calibrated in the modified arrangement by minimizing the slope deviations of a perfectly flat surface as that of a calm body of water (see Section 2.6), keeping fixed the parameters listed in Table 1 to the value measured with the Total Station; here *x _cj_* and *z _cj_* are two of the three coordinates in LabRF of the lens center of the j-camera.

In order to minimize the uncertainty, the full set of RR specimens (5 inner + 5 outer) was measured in the same session with the scan-parameters reported in Table 2; here the parameter *x*4 *set* is the value of *x _motor_* to which move the motorized rail to observe the center image of camera #1 on *x* = 0 of the chessboard used for the initial evaluation of position and attitude (see Figure 4). The introduction of *x*4 *set* is due to the unsatisfactory accuracy of the motorized rail initialization based on searching *x _motor_* = 0 by means of the limit switch detector; conversely the initialization made by the setting of *x*4 *set* is better than 0.1 mm.

**Table 1.** Parameters measured by the Total Station. PSA=point source array. The error is 0.3 mm for coordinates and 0.1 deg for angles.

Parameter	Experimental value	unit
Center of PSA	(−760.0 , 0.0 , 1483.7)	mm
Yaw Pitch Roll of PSA	(0.0 ,−30.6 , 0.0)	deg
Δ *x*_21_ = *x*_*c*2_ − *x*_*c*1_	-3.2	mm
Δ *z*_21_ = *z*_*c*2_ − *z*_*c*1_	1.6	mm

**Table 2.** Main VISproPT parameters used to measure the round-robin specimens.

Parameter	Inner	Outer
*x*_*max*_ (mm)	1960.0	2070.0
*x*_*min*_ (mm)	340.0	110.0
*x*_*step*_ (mm)	10.0	10.0
*x*4 *set*	1095.6	1095.6
Gray threshold	50	55

**Figure 8.**
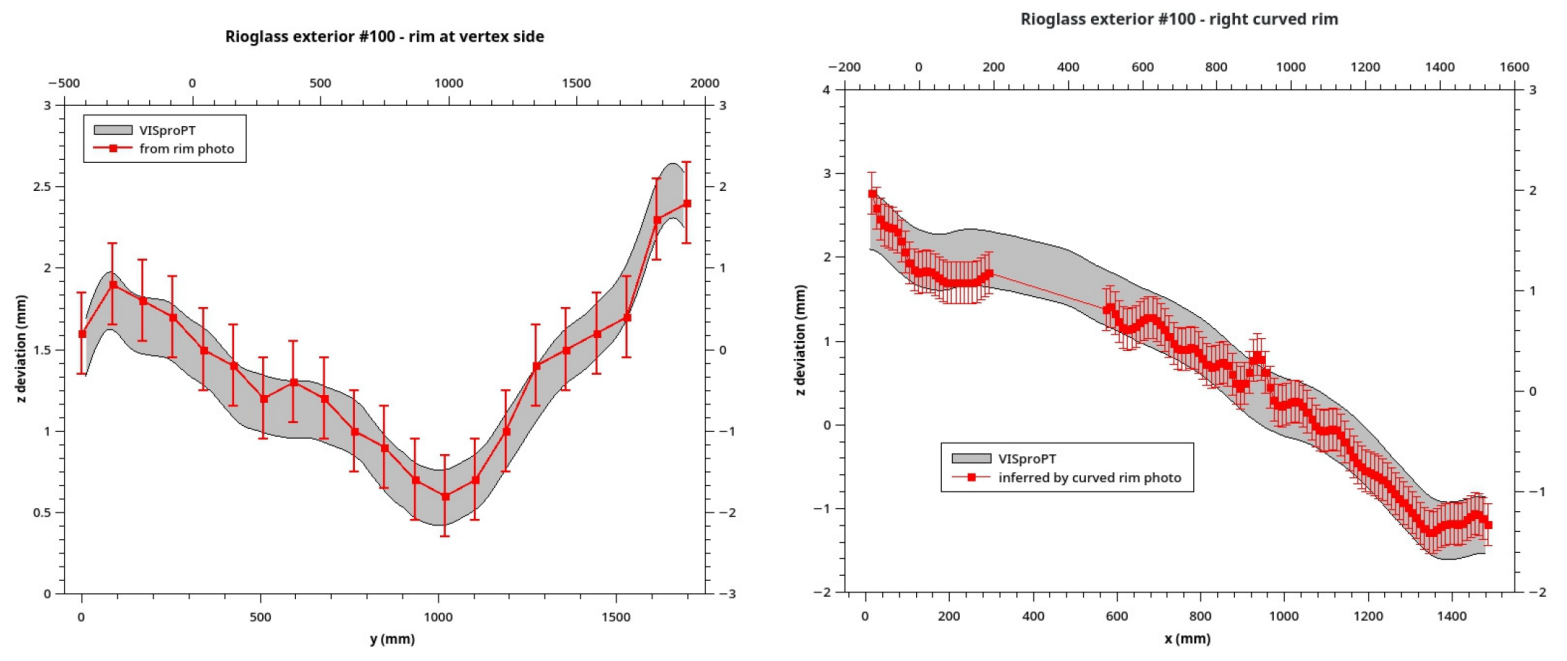
*z* deviation at two rims of Outer#100 obtained by VISproPT (grey band) and photograph (red error-bars).

The validation of the VISproPT results with those obtained with another independent technique is difficult. The most immediate solution might seem the Total Station, but unfortunately its Corner Cube Reflector accessory ^10^ cannot be placed freely on the surface because its weight-force deforms the panel surface itself, altering the result. Another approach, completely safe but limited, is the evaluation of the rim-shape by the analyses of its photography acquired by the side. Because of the VISproPT collocation in the Lab Hall, only two rims of the panel can be properly photographed. We use a Nikon D800 equipped with a Nikkor 24-120mm f3.5-5.6D. In each photography, the rim was manually sampled by reading the pixel-coordinates in several points, then transformed in millimeters by scaling with the total length of the rim, or the chord for the curved one. Finally the deviation from the ideal profile (linear for the edge close to the vertex and parabolic for the other) was calculated and compared with the ones obtained by VISproPT: as shown in Figure 8 the agreement is perfect although the VISproPT data-deviation refer to a section 10 mm far from the rim. Please note that, because of the shadowing by one of the four columns of the VISproPT structure, a small part of the curved rim is not visible; there deviation-data by photograph are missing.

**Figure 9.**
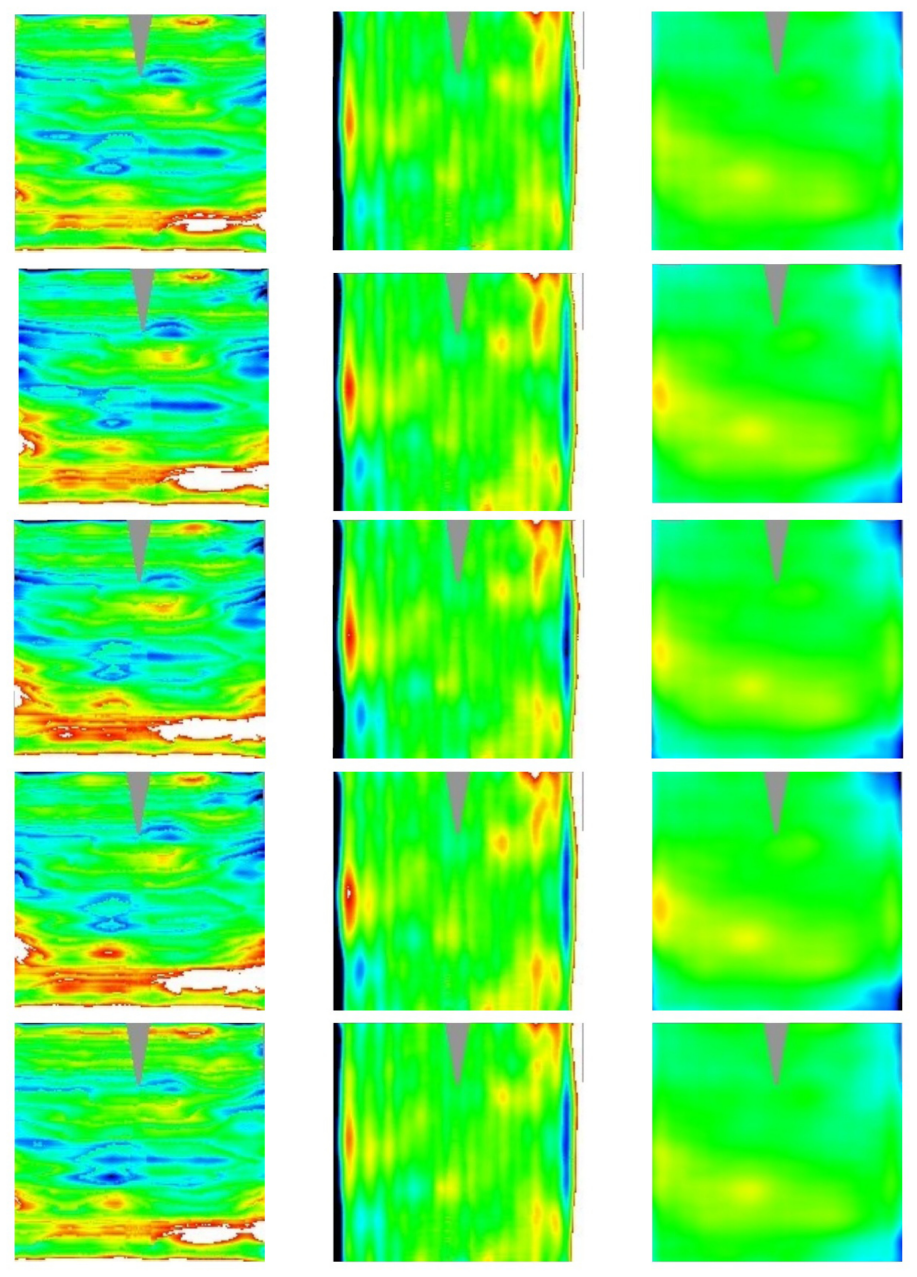
2D contour-maps of Δd *z*/d *x* (1st column), Δd *z*/d *y* (2nd column), and Δ *z* (3rd column) of the inner panels #58, #59, #60, #61, and #62 (sorted by rows). Color-range: [blue,red]= [-5,+5] mrad or [-2, 2] mm; black (white) below (above) that range; gray not sampled.

**Figure 10.**
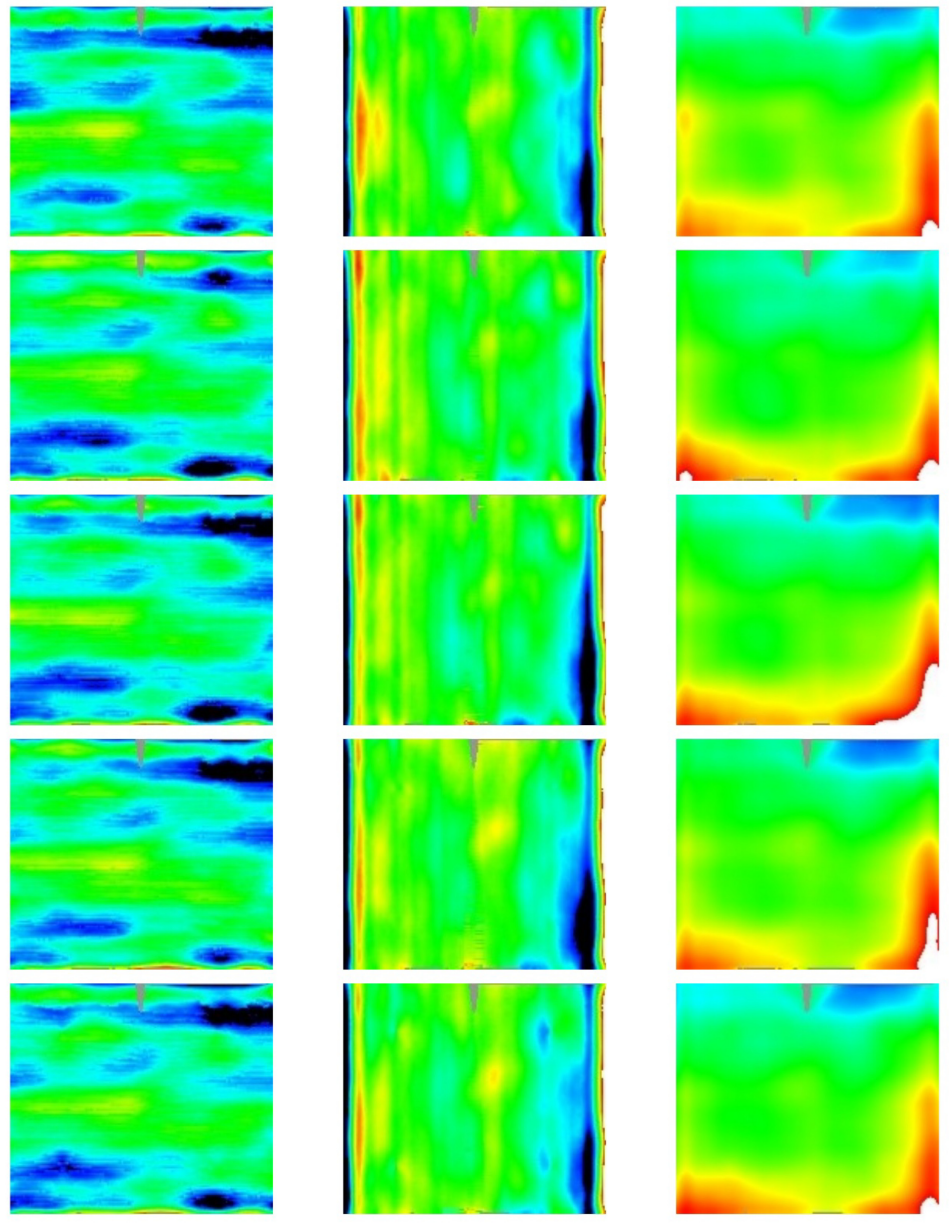
2D contour-maps of Δd *z*/d *x* (1st column), Δd *z*/d *y* (2nd column), and Δ *z* (3rd column) of the outer panels #93, #97,#99, #100, and #101 (sorted by rows). Color-range: [blue,red]= [-5,+5] mrad or [-2, 2] mm; black (white) below (above) that range; gray not sampled.

In order to show the instrument effectiveness, in the following the results we achieved on the RR specimen set are reported. In particular, the results of the 3D shape measurement are here reported as deviation from the ideal values (see Section 2.8): Table 3 reports the RMS value of the deviation, while Figure 9 and Figure 10 show the 2D contour-maps, respectively for inner and outer panel. Those 2D contour-maps are directly built using the multidimensional array S[j][i][k] introduced in Section 2.7 by assigning at the pixel of the map with coordinates [j][i] the color from blue to red proportionally to the experimental value in the range [-5,+5] mrad and [-2, 2] mm for Δd *z*/d *x* or Δd *z*/d *y* and Δ *z*, respectively; in white (black) the value above (below) the considered range; in gray the not sampled area.

**Table 3.** Root mean square deviation from the ideal shape.

Panel	Δd *z*/d *x* (mrad)	Δd *z*/d *y* (mrad)	Δ *z* (mm)
Inner#58	2.1	2.7	0.40
Inner#59	2.1	2.7	0.40
Inner#60	2.6	2.7	0.47
Inner#61	2.6	2.7	0.49
Inner#62	2.1	2.7	0.40
Outer#093	1.6	2.1	0.71
Outer#097	1.6	2.0	0.68
Outer#099	1.6	2.1	0.79
Outer#100	1.6	2.3	0.70
Outer#101	1.5	2.1	0.73

The panels belonging to the same kind (inner and outer) resulted to be affected by very similar deviations; probably they are a consequence of the good systematic nature of the production process which is affected by some imperfections. Once the 3D shape of the panel is known, one can evaluate the intercept factor, i.e. the fraction of the reflected radiation which is geometrically intercepted by the receiver. The computation is made by considering the standard value of the solar radiation divergence, 4.7 mrad (half-apex angle) and three different values for the longitudinal incidence angle ( *θ _L_*): 0, 35 and 70 deg.

**Figure 11.**
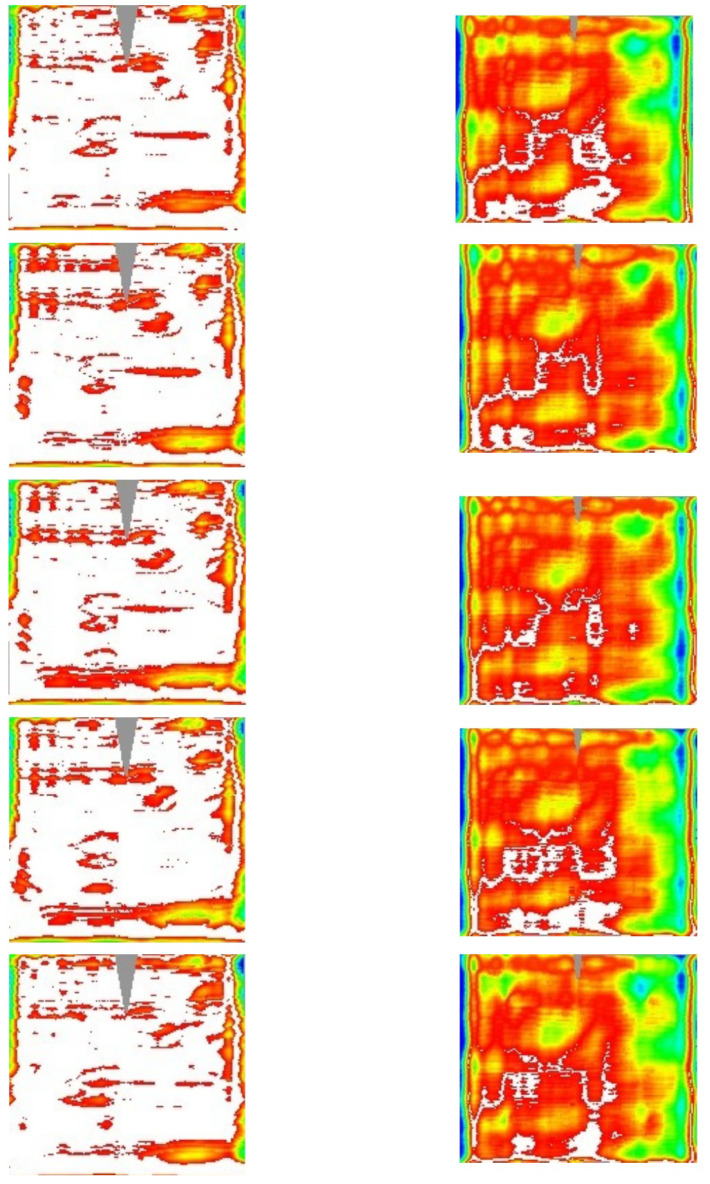
2D contour-maps of intercept factor at *θ _L_* = 70° for inner (left) and outer (right) panels, sorted by rows. Color-range: [blue,red] = [0,1); white full interception; gray not sampled.

Table 4 summarizes the intercept-factor mean-value, while Figure 11 shows the intercept factor as 2D contour-map at 70 deg; here white color is assigned for full interception, while colors from blue to red are used to index values from 0 to 1.

**Table 4.** Mean intercept factor.

Panel	*θ*_*L*_ = 0 ^°^	*θ*_*L*_ = 35 ^°^	*θ*_*L*_ = 70 ^°^
Inner#58	1.000	1.000	0.976
Inner#59	1.000	0.999	0.969
Inner#60	0.999	0.999	0.966
Inner#61	0.999	0.998	0.966
Inner#62	1.000	1.000	0.973
Outer#093	0.993	0.982	0.826
Outer#097	0.998	0.994	0.844
Outer#099	0.993	0.987	0.825
Outer#100	0.992	0.985	0.824
Outer#101	0.993	0.983	0.815

As expected the intercept factor mainly depends on Δd *z*/d *x* deviation, but for outer panels, at oblique incidence, also Δd *z*/d *y* deviation reduces the intercept factor.

## 4 Conclusions

The VISproPT instrument is the engineered version of the older VISprofile ^6^. The hardware was manufactured by MARPOSS Italia Spa ^7^ and funded by SFERA-III EU project ^8^, while ENEA developed the image processing software as well as the calibration procedure.

Differently from VISproLF ^5^, the instrument is not completely self-calibrating because some components have to be aligned and positioned with high accuracy; to that aim we used a Total Station.

The instrument is designed for indoor measuring the 3D shape of PT panels, but the outlined image processing method is also effective for differently curved specimens. As an example, in the current configuration, VISproPT can also measure flat specimens, like those for linear Fresnel plants, as long as their length does not exceed 1 m about; however this limit could be easily overcome by improving the hardware to accomplish the scan by only moving the specimen, keeping source point array and cameras in steady position.

The fine-calibration of the instrument is based on the shape measurement of a calm body of water. After that, the instrument accuracy resulted to be better than 0.1 mrad and 0.3 mm (RMS value over an area 1.2×0.8 m ^2^), respectively for slope and height of the surface. In a next paper dealing with the results of the round-robin, the accuracy of all the adopted instruments will be compared and discussed.

The VISproPT was exploited by measuring ten panels (5 inner + 5 outer) used in the round-robin (RR) on 3D shape measurements organized in the framework of the SFERA-III EU project, with the participation of F-ISE, DLR, NREL and SANDIA. The scansion of a specimen and the following image-processing typically take less than 90 s by a PC with Intel Xeon CPU E5-1620 v4 @ 3.50GHz; the duration of the measurement would be shorter using a more modern and performing PC.

For the sake of RR reliability, ENEA developed a simple supporting system which was proved to ensure a satisfactory reproducibility of the specimen-placing; supporting system and instructions will circulate together with the panels.

The deviation from the ideal parabolic profile are quite similar among the panels belonging to the same type, inner and outer; typically the slope deviation is better than 3 mrad and 2 mrad, respectively for inner and outer. The intercept factor at *θ _L_* = 0° is better than 99.9% and 99.2%, respectively for inner and outer kind.

## Ethics and consent

Ethical approval and consent were not required.

## Data Availability

Zenodo: mmonty1960/VISproPT: v2 http://doi.org/10.5281/zenodo.7889928 ^15^. This project contains the following underlying data as part of the software repository: RGinterior#58.7z (7zip-file containing the sequences of images acquired by the two cameras of the VISproPT for the exemplary inner PT panel #058). AcamNew.txt (setting-file with the instrument parameters used for measuring all the inner panels). Data are available under the terms of the GNU General Public License version 3.

## Software availability

Source code available from: https://github.com/mmonty1960/VISproPT

Archived source code at time of publication: http://doi.org/10.5281/zenodo.7889928 ^15^

License: GPL-3.0-only

## References

[1] Montecchi M : Italian Patent. RM2008A000151,2008.

[2] Montecchi M Benedetti A Cara G : Optical alignment of parabolic trough modules. 2010. Reference Source

[3] Montecchi M Casa MD : Post-assembly in-situ check of parabolic trough modules by VISshed. *AIP Conf Proc.* 2018;2033:030009. 10.1063/1.5067025

[4] Montecchi M Cara G Benedetti A : Visdish: A new tool for canting and shape-measuring solar-dish facets. *Rev Sci Instrum.* 2017;88(6):065107. 10.1063/1.4984944 28667978

[5] Montecchi M Cara G Benedetti A : Visprolf: Self-calibrating instrument for measuring 3d shape of linear fresnel facets. *Rev Sci Instrum.* 2020;91(8):083109. 10.1063/5.0013116 32872943

[6] Montecchi M Benedetti A Cara G : Fast 3d optical-profilometer for the shape-accuracy control of parabolic trough facets. 2011. Reference Source

[7] Marposs italia spa. 2023. Reference Source

[8] Sfera-iii, solar facilities for the european research area. 2023. Reference Source

[9] Solarpaces task iii:Solar technology and advanced applications. 2022. Reference Source

[10] Leica total station tda5005. 2022. Reference Source

[11] Qt: cross-platform application development framework for desktop, embedded and mobile. 2019.

[12] Open source computer vision library. 2019. Reference Source

[13] Frédéric Devernay: C/c++ minpack. 2017. Reference Source

[14] Montecchi M : Vispropt. 2022. Reference Source

[15] mmonty1960: mmonty1960/VISproPT: v2 (Version V2).Zenodo. [software],2023. 10.5281/zenodo.7889928

[16] Euler angles. 2020. Reference Source

[17] Ydrissi ME Ghennioui H Bennouna EG : A review of optical errors and available appli- cations of deflectometry technique in solar thermal power applications. *Renew Sustain Energy Rev.* 2019;116:109438. 10.1016/j.rser.2019.109438

