# VISproPT: A high-precision instrument for 3D shape analysis of parabolic trough panels

**DOI:** 10.12688/openreseurope.15967.2

**Published:** 2023-11-28

**Authors:** Marco Montecchi, Giuseppe Cara, Arcangelo Benedetti

**Affiliations:** 1 Solar Thermal, Thermodynamic and Smart Network Division, ENEA, ENEA-Casaccia via Anguillarese 301, Rome, 00123, Italy

**Keywords:** Concentrating Solar Power, parabolic-trough, reflective panels, 3D shape, optical profilometer

## Abstract

In Concentrating Solar Power plants, reflective panels are used to redirect solar radiation towards a receiver. Because the panel shape drives the radiation distribution around the focus where the receiver is placed, the 3D measurement is fundamental to assess the panel shape quality. The VISproPT instrument is the advanced version of the prototype VISprofile; these instruments are designed for indoor measuring of the 3D shape of parabolic-trough reflective panels. The VISproPT hardware has been manufactured by MARPOSS Italia Spa and funded by EU project ’Solar Facilities for the European Research Area - Third Phase’ (SFERA-III), while the image processing software as well as the calibration procedure, based on the measurement of a perfectly flat surface like that of a calm body of water, have been developed by the Italian National Agency for New Technologies, Energy and Sustainable Economic Development (ENEA). The instrument precision is better than 0.1 mrad and 0.3 mm (root mean square value over an area 1.2×0.8 m ^2^) for slope and height of the surface, respectively. Technical details of experimental set up and calibration-procedure are here reported. The instrument effectiveness is shown by reporting the results obtained on a set of 10 parabolic-trough specimens (5 inner + 5 outer) adopted to run the SFERA-III WP10 Task3 round-robin on 3D shape measurements among the different instruments used by the Fraunhofer Institute for Solar Energy Systems ISE (F-ISE), Deutsches Zentrum Fuer Luft - und Raumfahrt EV (DLR), the National Renewable Energy Laboratory (NREL) and Sandia National Laboratories (SANDIA).

## Plain language summary

The VISproPT is a novel instrument useful for scientists and technicians dealing with reflective panels for parabolic trough (PT) Concentrating Solar Power (CSP) plants. The VISproPT is an advanced laboratory instrument with high precision, designed to verify the shape compliance of PT panels. Deviations of slope and height from the ideal parabolic profile are evaluated and presented in terms of 2D contour maps as well as root mean square (RMS) values; these geometrical parameters are very useful to improve the production technology. Concerning the panel effectiveness in redirecting the solar radiation onto the linear receiver tube, the most representative parameter is the so-called *intercept-factor* (I-F), i.e. the geometrical intersection of the reflected solar beam with the receiver tube; the VISproPT software evaluates the I-F by considering the standard divergence of the solar radiation on the Earth (4.7 mrad half-apex angle) and the proper values of parabola focal-length and receiver-tube diameter; the I-F is reported as a 2D contour map and its mean value is calculated.

## 1 Introduction

The Visual Inspection System (VIS) approach was patented by ENEA in 2008 ^1^; it is based on the idea of placing a light source nearby the focus of the reflector and acquiring a number of images in the near-field from different positions of camera or source. On the basis of the VIS approach we developed the following instruments:

1. VISfield to verify the mutual optical alignment between receiver tube and parabolic trough reflector for modules in field ^2^
2. VISshed, the adaptation of VISfield for the quality control in the shed, soon after the module assembling ^3^
3. VISdish for facet-canting and 3D shape measurements of solar dish in field ^4^
4. VISproLF for 3D shape measurements of Linear Fresnel panels in laboratory/industry ^5^
5. VISprofile for 3D shape measurements of parabolic trough panels in laboratory

The latter instrument was developed in 2009 at ENEA Casaccia just as a demonstrative experimental set-up, alternative to the older 3D optical profilometer ^6^; the VISprofile was scarcely engineered and unsuitable for industry. On the other hand, shape/slope deviations of the reflective surface from the ideal profile cause solar radiation leakage with a reduction of efficiency. That makes the shape quality check a very important task. Therefore, since 2012, under the ENEA guidance, the Italian worldwide leader in Measurement, Inspection and Testing, MARPOSS ^7^ started to engineer a new version of the instrument, named VISproPT, suitable for commercial purposes. The prototype was soon ready but because of weakness of CSP market and insufficient ENEA funding, it remained in stock until 2018 when it was delivered to ENEA Casaccia to be acquired under the umbrella of the EU SFERA-III project ^8^. The hardware commissioning was delayed to September 2021 because of the COVID-19 pandemic and the resulting shortage of electronic components. Only from that time the development of the image processing software as well as of the calibration procedure could start. The good side of such a long gestation is the relevant progress, matured in the meanwhile, of the ENEA knowhow on the use of digital cameras in 3D geometric characterization; as a result now the instrument calibration is much simpler than what initially prefigured.

To exploit the new instrument we took the opportunity of the WP10 Task3 in the SFERA-III project ^8^ to launch the proposal of a new round-robin (RR) on 3D shape measurements of panels for PT solar collectors; a previous attempt, accomplished about 10 year ago in the SolarPACES TaskIII framework ^9^, did not give satisfactory results because the differences among the results obtained by the participants were greater than the experimental error; as a matter of fact, today a dedicated guideline on the topic is still missing. The proposal was accepted by F-ISE and DLR and then was extended to the National Renewable Energy Laboratory (NREL) and Sandia National Laboratories (SANDIA), which benefit of the Transnational Access tools offered by SFERA-III to send teams over Europe for visiting research infrastructures and participating in the introductory meeting to the round robin itself.

At the time of writing this article, the round-robin has just started; the global results will be published in a next future. This paper is focused on the novel instrument VISproPT; the results achieved by ENEA are here presented just to illustrate the instrument functionality.

## 2 VISproPT basis

### 2.1 Main hardware components

In general, the VIS method ^1^ just needs a good monochrome digital camera and a structured light source; the source type and the arrangement of these components around the object to be measured depend on the specific case.

The engineered version of the VISprofile is named VISproPT to distinguish it from the other instrument, named VISproLF ^5^, designed for 3D shape measurements of panels for linear Fresnel collectors.

**Figure 1.**
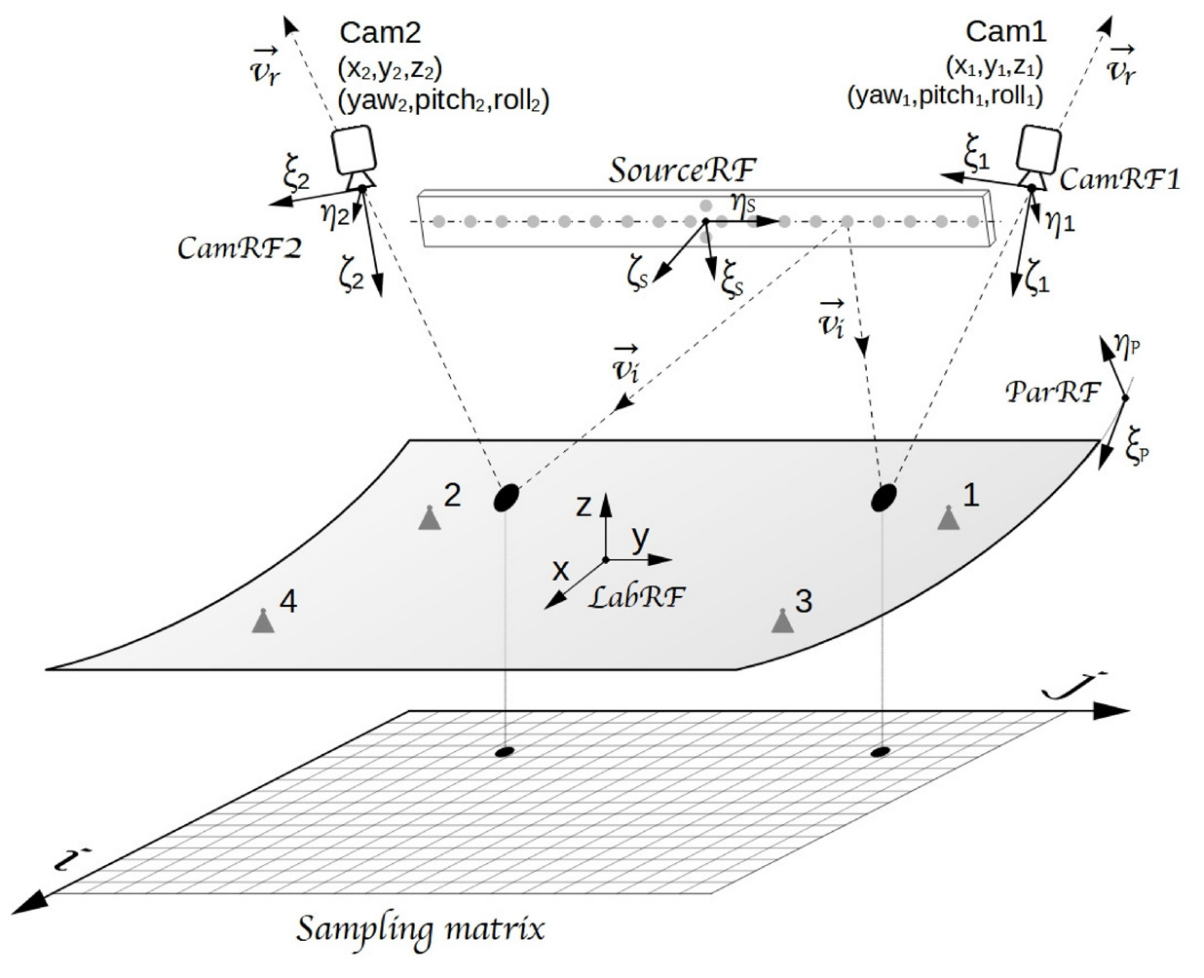
VISproPT optical sketch: two digital cameras (Cam1 and Cam2) acquire photos of the panel surface with the reflected images of the point source array. Five reference frames (RF) are used in the image-processing: 1) parabola ( *ParRF*), 2) laboratory ( *LabRF*), 3) point source array ( *SourceRF*), 4) Cam1 ( *CamRF1*), 5) Cam2 ( *CamRF2*). At the end of the image processing, the experimental values of height and partial derivatives of the surface are gridded over the *sampling matrix*.

**Figure 2.**
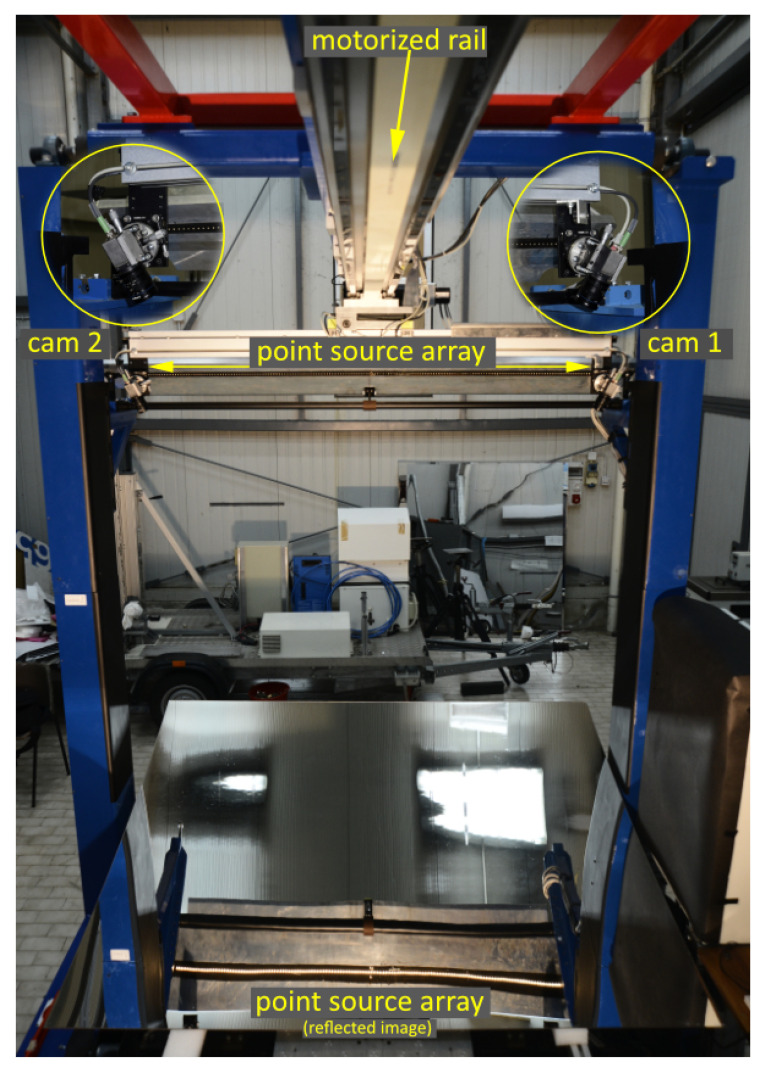
VISproPT hardware. The main components are: the point source array, two digital cameras and a motorized rail for moving the cameras over the specimen.

The optical sketch adopted in VISproPT is shown in Figure 1, while the hardware, manufactured by MARPOSS Italia Spa ^7^, is shown in Figure 2. The PT panel to be investigated is placed on 4 supports (1,2,3,4) aligned on the same horizontal plane and positioned accordingly to the panel design. The origin of the laboratory reference frame *LabRF* is approximately in the center of the 4 attaching points, with the Z axis aligned along the vertical and the X axis parallel to the motorized rail (and the direction of curvature of the panel).

A motorized rail is placed horizontally at *z* ≈ 1.5 m in *LabRF*; its horizontal alignment is optimized by a Total Station Leica TDA 5005 ^10^. Two digital cameras are housed on the motorized rail cart. The rail is long enough (about 3 m) to obtain that the point source images, reflected by the panel and viewed by the cameras, scan the entire panel surface during the linear translation of the cameras along a suitable range [ *M _min_*, *M _max_*], where *M* is the cart position on the rail given by the encoder.

The light source is composed by an off-the-shelf low-cost Lighting Emitting Diode (LED) strip wrapped in a metallic box of which one face, made of an aluminum thick plate, has been precisely drilled by means of a computer numerical control machine; that forms our linear array of point sources with step 10.00 mm. Thanks to the chosen materials and to the specific design, the array of point sources can be assumed as perfectly linear with accuracy better than 0.01 mm for each point, in any of the three dimensions.

The central point of the array is marked by a couple of point sources symmetrically displaced (10.00 mm) from the array central-line; as will be explained later, such detail is fundamental to allow the proper attribution of the origin point source for each of the reflected-point images displayed in the images captured by the cameras.

By means of the Total Station the point source array is aligned parallel to the Y axis, and the position of its center is measured.

Concerning the digital camera, the instrument makes use of two identical units composed by:

- camera: Baumer VCXG-51M, 2448 px x 2048 px complementary metal-oxide semiconductor (CMOS) sensor, monochrome
- objective: Opto Engineering EN5MP0816 5 Megapixel 8 mm 1:1.6 2/3”

The two cameras are fastened on a rod attached to the cart of the motorized rail; the rod is horizontal and parallel to the Y axis. The adoption of two cameras allows to keep the width of the scanned area constant during their linear translation; to that purpose each camera is positioned at about the same y-coordinate of the underlying curved-rims of the PT panel; the cameras are oriented in such a way to frame the entire width of the panel (in the flat direction) with the widest side of the CMOS sensor. Thanks to the procedure described in Section 2.5, the two cameras can be positioned and oriented without paying particular attention.

To prevent changes induced by thermal expansion, the laboratory hall is equipped with several heat pumps working to maintain 23 °C all the time; the humidity is not explicitly controlled, even if the cooling system avoids the occurrence of excesses. During the instrument development, in different periods of the year, the position of the main parts of the instrument was periodically measured by the total station, but we never observed deviations greater than the experimental error (0.3 mm). Anyway, as discussed in Section 2.6, after any set-up modification, the final calibration must be run, thus many VISproPT parameters are newly optimized.

To the author’s knowledge the VISproPT instrument is totally original for both hardware configuration (the optical sketch) and the approach adopted to evaluate the 3D shape of PT panels.

### 2.2 Software

The hardware provided by MARPOSS includes an industrial computer hosting a software for the basic instrument management, with commands related to the cart position *M* on the rail:

- go home and set *M* = 0
- go to *M*
- acquire a single image (one for each camera) at the given cart position *M*
- scan the panel surface from *M _max_* to *M _min_*, acquiring images with step *M _step_*

This MARPOSS software is not provided as part of this article because it is proprietary; on the other hand, it is strictly related to the hardware components adopted in the VISproPT, and not useful for others. In other words, if the reader wanted to develop a similar instrument, he would have to write his own software equipped with the libraries necessary for managing the chosen hardware components (image acquisition by the cameras and motorized-rail handling). Thus the omission of the MARPOSS software does not limit the readers from replicating the methods and data processing described in this article.

The development of the software for instrument-calibration and image-processing was totally demanded to ENEA which holds the full know-how of the VIS method. For the sake of simplicity this software is hosted on the Lab PC from which the instrument can be controlled by sending command-scripts to the industrial computer. As a drawback, the sequence of acquired images must be transferred to the Lab PC before they can be processed; in the future, the new software could be directly installed in the industrial computer.

The main features of the software developed by ENEA are:

1. C++ written;
2. provided with a Qt graphical user interface ^11^;
3. based on the OpenCV library ^12^ which includes a rich tool-set for computer vision;
4. equipped with nonlinear least squares offered by CMINPACK library ^13^.

This software covers all the steps of 3D shape measurements:

- Camera-lens calibration, for image undistortion
- Instrument calibration
- Image-processing for evaluating: i) 3D shape (slopes d *z/*d *x*, d *z/*d *y* and height *z*), ii) deviations from the ideal shape and, last but not least, iii) evaluation of the intercept factor at a given longitudinal angle.

To add value to the work done so far, believing in knowledge sharing, we took the opportunity offered by Open Research Europe to make the VISproPT software available for everyone as an open source software under the GNU General Public License as published by the Free Software Foundation version 3. Code source files and data of one exemplary measurement can be freely downloaded from *Software availability* and *Data availability* ^14, 15^.

Although already described in 5, in order to make the image processing we developed for VISproPT fully understandable, the next three paragraphs shortly recall some well assessed concepts, such as: pinhole camera model, camera calibration, transformation rules between two reference frames, and determination of position and attitude of the camera.

### 2.3 Pinhole camera model and camera calibration

**Figure 3.**
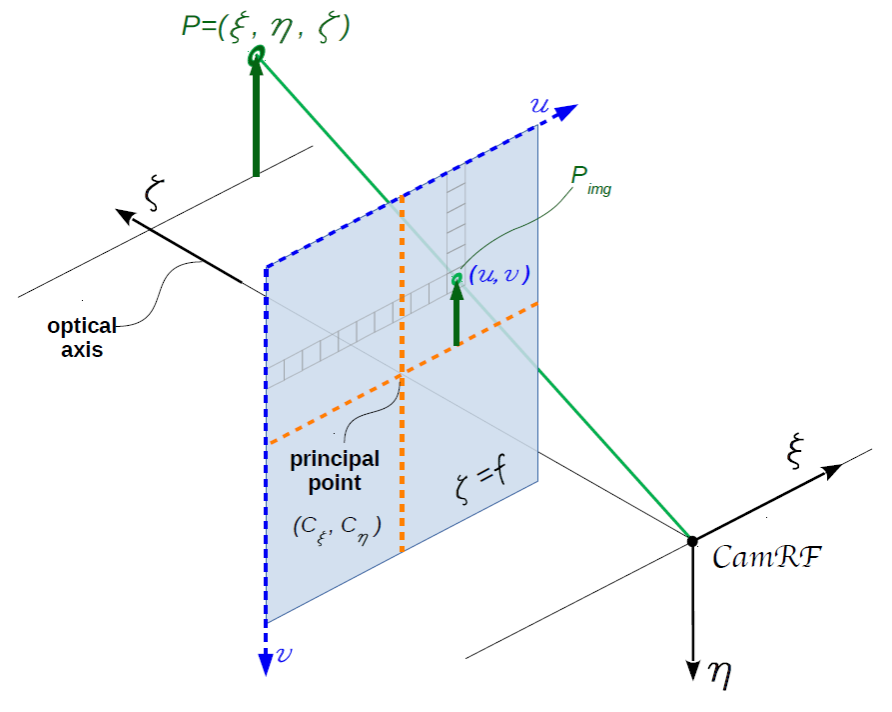
Pinhole camera model. Source point *P*, image point *P _img_*, and lens center (the origin of the camera reference frame *CamRF*) are aligned along the same straight line.

The *pinhole camera model* ^12^, shown in Figure 3, is commonly used for 3D reconstruction from 2D images. Let us consider the camera reference frame ( *CamRF*) depicted in the figure: the 3D point *P* = ( *ξ*, *η*, *ζ*) is imaged in the point *P _img_* = ( *u*, *v*) on the sensor (commonly, CCD or CMOS) which is set on the plane *ζ* = *f* orthogonal to the optical axis, i.e. the *ζ*-axis; the origin of *CamRF* is the *lens center*; the *ζ*-axis crosses the sensor at the *principal point* with pixel coordinates ( *c _ξ_*, *c _η_*); the sensor is unrealistically set between lens-center and object to take into account the image straightening provided by the sensor electronic; the *ξ*-axis ( *η*-axis) is parallel to sensor rows (columns). In the pinhole camera model, source ( *P*), image ( *P _img_* ) and lens-center (the origin of *CamRF*) are on the same straight line, thus

$$u=f_{\xi }\frac{\xi }{\zeta }+c_{\xi }\;(1)$$

$$v=f_{\eta }\frac{\eta }{\zeta }+c_{\eta }\;(2)$$

where *f _ξ_* and *f _η_* are the focal lengths, and ( *c _ξ_*, *c _η_*) are the coordinates of the principal point; all these parameters are expressed in pixel units and stored in the so-called *camera matrix*. If the pixel is a perfect square, like for the here adopted cameras, *f _ξ_* = *f _η_* = *f* . It must be noted that the principal point is close to the sensor center, but generally it is not coincident with it.

Unfortunately any real lens usually induces some *distortions* (mostly radial) in the image. In order to make the pinhole camera model valid, the image should be first *undistorted* on the basis of *camera matrix* and *distortion coefficients*; those are obtained by calibrating the *camera-lens* system; please note that the evaluation of focal length *f* and principal point ( *c _ξ_*, *c _η_*) is part of that calibration. Noteworthy, camera matrix and distortion coefficients are strictly related to the specific camera-lens system, so that even if only the focus is slightly changed, the whole calibration procedure shall be repeated.

Procedure and functions for camera calibration and image correction are part of the rich OpenCV library ^12^ to which we refer the reader for further information. Here we just recall that calibration is obtained by processing a sequence of images of the same chessboard in different orientation. Such a chessboard has to be printed and stitched on a rigid flat substrate by the user. We used a black and white chessboard composed by 54 × 46 squares with 10.0 mm side each.

### 2.4 Transformation rules

As shown in Figure 1, five reference-frames play an important role in VISproPT:

- the first two, named *CamRF1* and *CamRF2*, are related to the cameras (as detailed in Figure 3)
- the third is the Laboratory frame ( *LabRF*), which origin is set in the center of the four attaching points, with the *z*-axis aligned along the vertical, and the *x*-axis parallel to the motorized rail
- the fourth reference-frame, named *SourceRF*, is related to the point source array: it is centered between the two special central points, with the y-axis along the array, and z-axis orthogonal to the drilled-face of the metallic box
- the fifth ( *ParRF*) is the natural reference frame of the ideal parabola, where $\eta =\frac{1}{4f_{p}}\xi^{2}$ and *f _p_* is the parabola focal length

The 3D coordinates of a point in one of these reference-frames can be expressed in those of any other by translation and rotation. The translation rules are well known; conversely for rotations there are dozens of different conventions, depending on the considered angles and the sequence of rotations around the axes ^16^. Among them we chose the one normally used for aircraft, where the 3 rotation angles, named *Yaw*, *Pitch* and *Roll*, are associated to the axes as follow: Yaw → *ζ*, Pitch → *η* and Roll → *ξ*. The rotations must be applied in such order.

The transformation rules of the coordinates ( *ξ*, *η*, *ζ*) from *CamRF*, *SourceRF* or *ParRF* (the plane) to *LabRF* ( *x*, *y*, *z*) (the Earth) are

$$\begin{array}{l} \;x=c_{1}c_{2}\xi +(c_{1}s_{2}s_{3}-c_{3}s_{1})\eta +(s_{1}s_{3}+c_{1}c_{3}s_{2})\zeta \\ \;y=c_{2}s_{1}\xi +(c_{1}c_{3}+s_{1}s_{2}s_{3})\eta +(c_{3}s_{1}s_{2}-c_{1}s_{3})\zeta \\ \;z=-s_{2}\xi +c_{2}s_{3}\eta +c_{2}c_{3}\zeta \end{array}\;(3)$$

where *s _j_* and *c _j_* are respectively sin and cos with argument *j* = 1 → Yaw, *j* = 2 → Pitch, *j* = 3 → Roll.

With the same symbolism, the opposite transformation rules from Earth ( *LabRF*) to plane ( *CamRF*, *SourceRF* or *ParRF*) are

$$\begin{array}{l} \;\xi =c_{2}c_{3}x-c_{2}s_{3}y+s_{2}z\\ \;\eta =(c_{1}s_{3}+c_{3}s_{1}s_{2})x+(c_{1}c_{3}-s_{1}s_{2}s_{3})y-c_{2}s_{1}z\\ \;\zeta =(s_{1}s_{3}-c_{1}c_{3}s_{2})x+(c_{3}s_{1}-c_{1}s_{2}s_{3})y+c_{1}c_{2}z \end{array}\;(4)$$

Equation 3 and Equation 4 represent only the rotation; the translation is applied after the rotation.

### 2.5 Position and attitude of cameras

**Figure 4.**
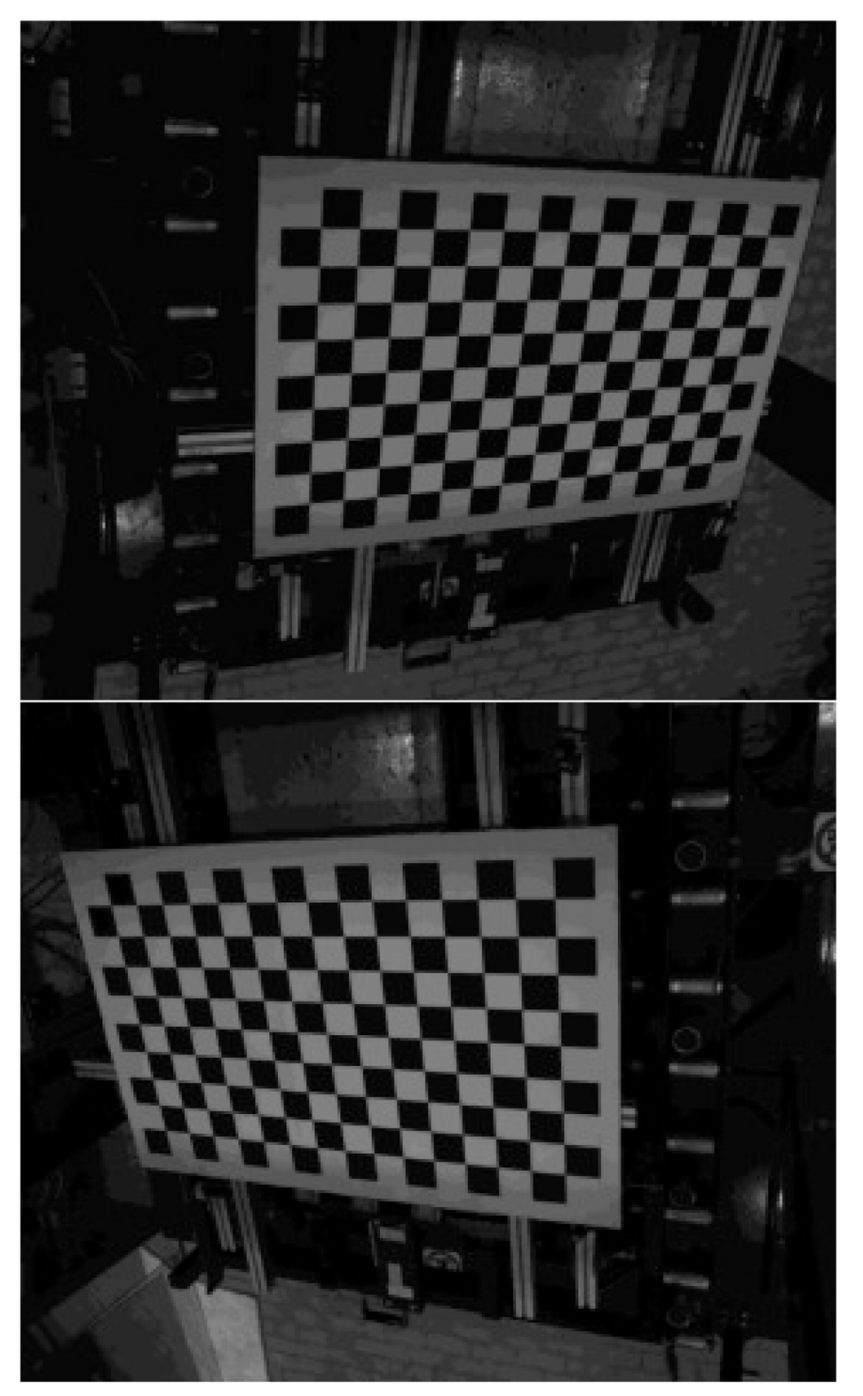
View of the chessboard with 15 × 9 = 135 corners from Cam1 (up) and Cam2 (down).

Position and attitude of each camera in *LabRF* are fundamental parameters. They can be easily obtained by analyzing the image of a chessboard placed on the specimen holder (see Figure 4). The chessboard is precisely positioned by means of the Total Station.

Our chessboard consists of 16 × 10 squares, resulting in 15 × 9 = 135 corners, i.e. the points where the corners of 4 squares (2 white and 2 black) are touching one to each other; the step along columns and rows is the same, 70.0 mm. In order to improve the measurement repeatability after any reboot, we introduce the parameter *M*4 *set*: it is the value of *M* to set the center image of camera #1 on *x* = 0 of the chessboard. As a matter of fact, the accuracy of the motorized rail initialization given by the command *go home and set M* = 0 is of several millimeter; conversely the initialization made by means of *M*4 *set* is better than 0.1 mm.

Each of the two images shown in Figure 4 is analyzed for evaluating the pixel-coordinates ( *u*, *v*) of each of the 135 corners in the proper *CamRF*. This job can be automatically accomplished by using the function findChessboardCorners of the OpenCV library ^12^.

On the other hand the coordinates of these corners can be easily computed in *LabRF* because the chessboard is: i) aligned on the horizontal plane defined by the *X Y* axes; ii) centered in the origin (0, 0, 0); iii) oriented with rows (columns) parallel to *Y* ( *X*) axis. Under these conditions the coordinate of the corner at row *i* = 0, 1, 2, .., *NR* − 1 and column *j* = 0, 1, 2, .., *NC* − 1 are

$$\begin{array}{l} x_{j,i}=(\frac{N_{R}+1}{2}-1-i)\text{Δ}\\ y_{j,i}=(\frac{N_{C}+1}{2}-1-j)\text{Δ}\\ z_{j,i}=0 \end{array}\;(5)$$

where Δ = 70.0 mm, *N _R_* = 9 and *N _C_* = 15.

When corner-coordinates, lens-center-position ( *x _c_* , *y _c_* , *z _c_*) and camera-attitude (Yaw,Pitch,Roll) are known in *LabRF*, the expected position of each corner in the acquired image can be evaluated by means of the pinhole camera model: in *LabRF* the corner ( *j*, *i*) is located at the end of the vector

$${\vec{v}}_{j,i}=(x_{j,i}-x_{c},y_{j,i}-y_{c},z_{j,i}-z_{c})\;(6)$$

applied to the camera lens-center. By means of Equation 4 the components of ${\vec{v}}_{j,i}$ in *LabRF* (given in Equation 6) can be transformed in *CamRF*, obtaining ( *ξ*_*j*, *i*_, *η*_*j*, *i*_, *ζ*_*j*, *i*_). Then, according to the pinhole camera model, the image of the corner ( *i*, *j*) is expected in

$${\vec{u}}_{j,i}=\frac{f}{\zeta_{j,i}}(\xi_{j,i},\eta_{j,i},\zeta_{j,i})=(u_{j,i}-c_{\xi },v_{j,i}-c_{\eta },f)\;(7)$$

where ( *u*_*j*, *i*_, *v*_*j*, *i*_) are its pixel coordinate.

The comparison between experimental and expected values of the corner pixel-coordinates is a powerful method for optimizing the values of ( *x _c_* , *y _c_* , *z _c_*) and (Yaw,Pitch,Roll). More precisely we adopted the modified Levenberg Marquardt non linear least square algorithm, offered by CMINPAK library ^13^ on the differences between calculated and experimental corner pixel coordinates, for an amount of 2×15×9 = 270 data. The best fit procedure is launched starting from a rough (but realistic!) initial evaluation of position and attitude of the camera. The procedure is separately applied to each camera.

### 2.6 Instrument calibration

Unfortunately we realized that the evaluation of principal point ( *c _ξ_*, *c _η_*) and focal length *f* by the chessboard method, explained in Section 2.3, is not precise enough for our purposes: we found that as the number of images considered for the camera calibration increases, the succession of estimated values does not seem to converge because at each step the result is affected by the portion of the visual field occupied by the chessboard in the new added image. This weak point has a cascade effect on the accuracy of the evaluation of lens-center position and camera-attitude described in Section 2.5.

To overcome this problem we enriched the software with the final instrument-optimization: principal point, focal length, lens-center position and attitude of each camera are optimized with the modified Levenberg Marquardt non linear least square algorithm, by minimizing the first derivatives d *z/*d *x* and d *z/*d *y* of the 3D shape measurement of a perfectly flat surface as that of a calm body of water; as explained in the next section, those first derivatives are part of the results stored in the sampling matrix S.

**Figure 5.**
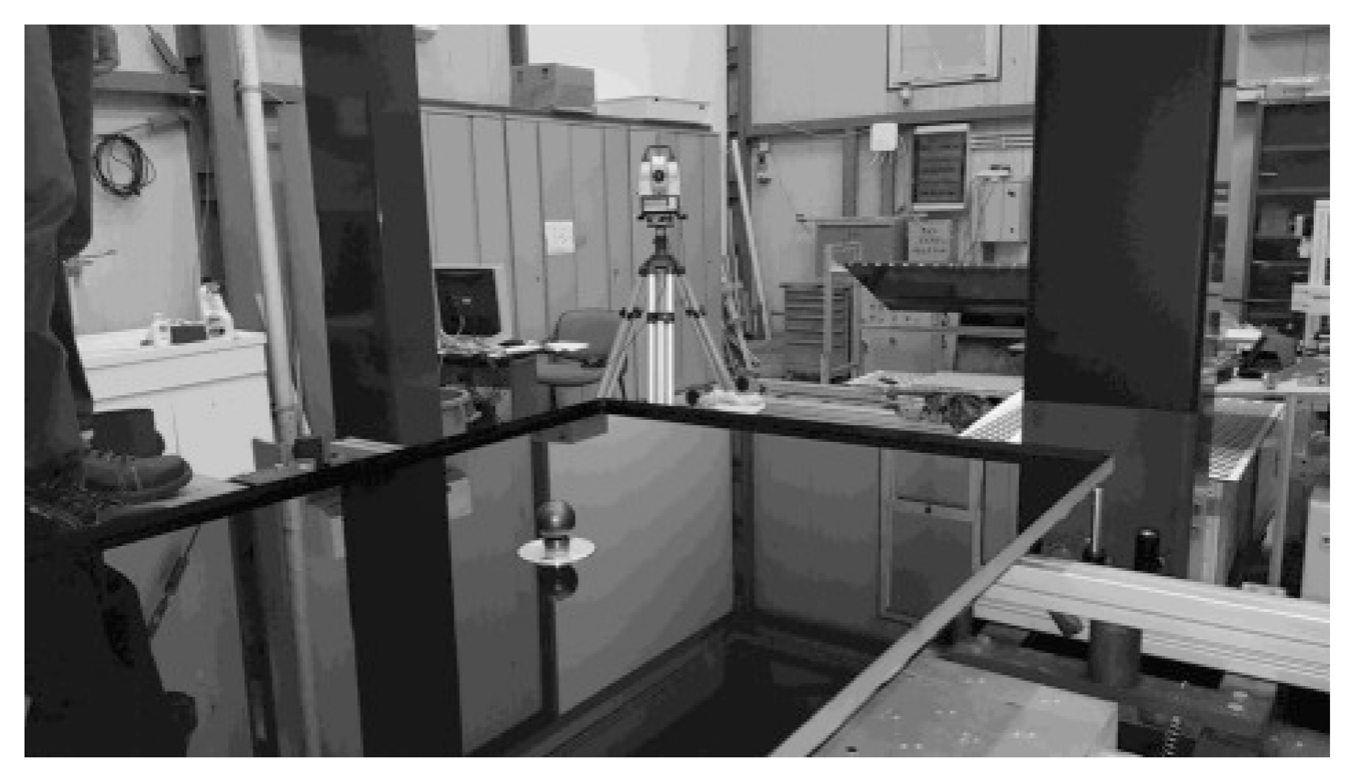
Basin with water and corner cube reflector accessory ^10^ for z measurement by Leica Total Station.

Thanks to the laboratory’s remoteness from busy streets, undergrounds and railways, once calm, the water surface remains totally unperturbed. As shown in Figure 5 a basin (1.2 × 0.8 m ^2^) is placed on the attaching points of the VISproPT; once again the z coordinate of the air|water interface was evaluated by means of the Total Station. Moreover, said *x _cj_* and *z _cj_* two of the three coordinates of the lens center of the j-camera in *LabRF*, the differences Δ *x*_21_ = *x*_*c*2_− *x*_*c*1_ and Δ *z*_21_ = *z*_*c*2_ – *z*_*c*1_ can be directly measured by the Total Station and used as constrains in the best-fit by setting *x*_*c*2_ = *x*_*c*1_ + Δ *x*_21_ and *z*_*c*2_ = *z*_*c*1_ + Δ *z*_21_; thus *x*_*c*2_ and *z*_*c*2_ are derived parameters.

After the instrument calibration, the measured shape of the water surface results to be flat for better than 0.1 mrad and 0.3 mm (RMS values), respectively for slope and height of the surface with approximated area of 1.2 × 0.8 m ^2^.

### 2.7 Evaluation of the 3D shape

Once lens-center position, camera-attitude and point-source-array deployment are known in *LabRF*, the instrument is ready to perform 3D shape measurements. This section describes the original procedure we are proposing.

The measurement is based on the analysis of the two sequences of images acquired by the two cameras along the translation *M _max_* → *M _min_*; the range of the translation is properly set to maximize the scan area of the specimen surface, but taking care to avoid any interruption of the sequence of the imaged point sources. As a matter of fact, avoiding discontinuity between one point source and its first neighbors is essential for the right association between imaged and origin point source, as will be clarified shortly.

**Figure 6.**
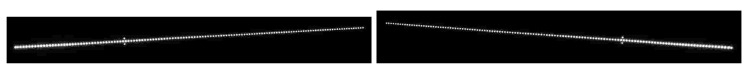
Point source array reflected by water and viewed by Cam1 (left) and Cam2 (right).

Differently from conventional deflectometry ^17^, VISproPT adopts a fully binary method. As an example Figure 6 shows the relevant part of a couple of images acquired for the instrument calibration. The images are monochrome with 8 bit pixel-depth, i.e. the luminosity ranges in [0, 255].

The automatic detection of the point sources contained in the image is fundamentally based on grey thresholding around a suitably set value (in this case 30): the luminosity of the pixels forming the point source image (technically named “blob”) is above the threshold, while all the surrounding pixels have lower luminosity. Anyway additional constrains, as minimal blob area, minimal distance from two blobs, etc, can be set if necessary. To avoid false blob-detection, the laboratory must be sufficiently darkened.

Initially, the software performs two operations for any image of each sequence:

1. automatic detection of the blobs framed in the image by means of the SimpleBlobDetector Class offered by the OpenCV library (the luminosity threshold is one of the input parameters)
2. for each detected blob, the corresponding values of camera-number, frame-number, origin-point-source-number and ( *j*, *i*) pixel coordinates of the blob centroid are stored in the array

double results[550000]; /*
results[i*6+ 0] = N. frame
results[i*6+ 1] = N. Camera
results[i*6+ 2] = N. source
results[i*6+ 3] = /
results[i*6+ 4] = column blob-coordinate (px)
results[i*6+ 5] = row blob-coordinate (px) */


where i is the progressive number of the detected blob, and N.source is the index of the point source from which the blob originates; the software takes care of the association


blob↔N.source

starting from the recognition of the special doublet marking the center of the point source array (see Figure 6); from that, the proper point-source index is sequentially assigned to all the surrounding blobs.

At the end of the images processing, the results array is populated with the data of all the blobs detected. Note that, unless the distortion coefficients are modified, the data stored in results do not depend on focal, central point, lens-center position and camera-attitude. This fact greatly reduces the computing time of the instrument calibration algorithm because the image-analysis does not have to be repeated at each iteration of the best-fitting.

Then the second stage of the processing starts; it consists in the use of the data already stored in results to evaluate the parameters of the surface at each point of reflection; the new parameters are computed and stored in the multidimensional sampling matrix displayed in Figure 1


double S[Nj][Ni][12]={}; /*
S[j][i][0] = vn[0] \
S[j][i][1] = vn[1] - normal unit vector
S[j][i][2] = vn[2] /
S[j][i][3] = z_ideal (height) (mm)
S[j][i][4] = N. of independent evaluations
S[j][i][5] = dz/dx (partial y-derivative)
S[j][i][6] = dz/dy (partial x-derivative)
S[j][i][7] = z_exp (height) (mm)
S[j][i][8] = dz/dx_ideal
S[j][i][9] = dz/dy_ideal
S[j][i][10]= intFat
S[j][i][11]= z_exp rms */


where the indices [j][i] give the cell position (j-column and i-row) of the sampling matrix drawn in Figure 1, while the third index is used to store the several parameters associated to the cell. Please note that the sampling matrix ensures the regular gridding of the final data in the plane XY of the *LabRF*.

More precisely the data-set of each blob stored in results is processed as follow:

- In *CamRF*, computing of the reflected unit vector $\vec{r}$ from the knowledge of the pixel coordinate of the blob ( *u*, *v*) and the central point ( *c _ξ_*, *c _η_*) of the camera-lens system (see Equation 7)
- Transformation of $\vec{r}$ in *LabRF* by Equation 3 using the knowledge of the lens-center coordinates and camera-attitude
- Calculation of the intersection between the straight line driven by $\vec{r}$ applied to the lens-center (with *x _c_* actualized to the proper frame-number) and the specimen surface represented by the *z*-data stored in S[j][i][7]; the knowledge of the reflection-point position allows determining the coordinate [j][i] of the cell containing it
- Calculation of the incidence unit vector $\vec{i}$ in *LabRF* from the knowledge of the position of the origin point source and the one of the point of reflection (as determined in the previous step)
- Calculation of the unit vector normal to the surface $\vec{n}$ ∝ –( $\vec{i}$ – $\vec{r}$ ) and, consequently, the partial derivatives along X and Y axis

We use to set the size of *S* matrix cell to the step-value used for the image acquisition during the camera translation, typically 10.0 mm. Generally each cell is filled with more than one point; the mean value of the normal unit vector is finally stored. Then the sub-region of *S* matrix really populated by experimental data is delimited and processed with an inpainting routine to fill the few cells not containing data.

Finally, 2D integration is accomplished starting from each of the attaching point (where *z* is known); then the mean value is stored in S[j][i][7] while the RMS value in S[j][i][11].

At the first run the panel-shape is assumed to be ideal, that is S[j][i][7]=S[j][i][3]; then the whole procedure is repeated by using each time the refreshed value of S[j][i][7] obtained by the 2D integration; when the maximum difference between one iteration and the following is less than a threshold value (as an example 0.03 mm) the loop ends. Generally the convergence is reached after a few tens of iterations.

Once the 3D shape of the panel is known, the intercept factor can be computed; the software considers the standard solar divergence of 4.7 mrad (half apex-angle) and the longitudinal incidence angle *θ _L_* set by the user.

As a final step the software builds and saves as Joint Photographic Experts Group (JPEG) images the 2D contour maps representing the deviation of d *z/*d *x*, d *z/*d *y*, height *z* and the intercept factor; here for *deviation* we refer to the difference between experimental and ideal values.

It is noteworthy that the above procedure is not restricted to parabolic trough panels: as a matter of fact it was successfully used to measure the water surface. Anyway the movement limited to the cameras, with the source in steady position, greatly limits the maximum size of the measurable area of flat specimens. On the other hand, the VISproPT hardware could be easily implemented by adding a linear translator to move the specimen along the *x*-axis, and scan the specimen with cameras and point source array in steady position. This alternative configuration could be useful for linear-Fresnel panels.

### 2.8 Ideal parabola reference frame

For evaluating the compliance of height *z* and slopes, d *z/*d *x* and d *z/*d *y*, observed in *LabRF* with the values expected for the ideal parabola, the knowledge of the relationships between *LabRF* and *ParRF* (where the profile is described by the canonical equation $\eta =\frac{1}{4f_{p}}\xi^{2}$ ) is fundamental.

**Figure 7.**
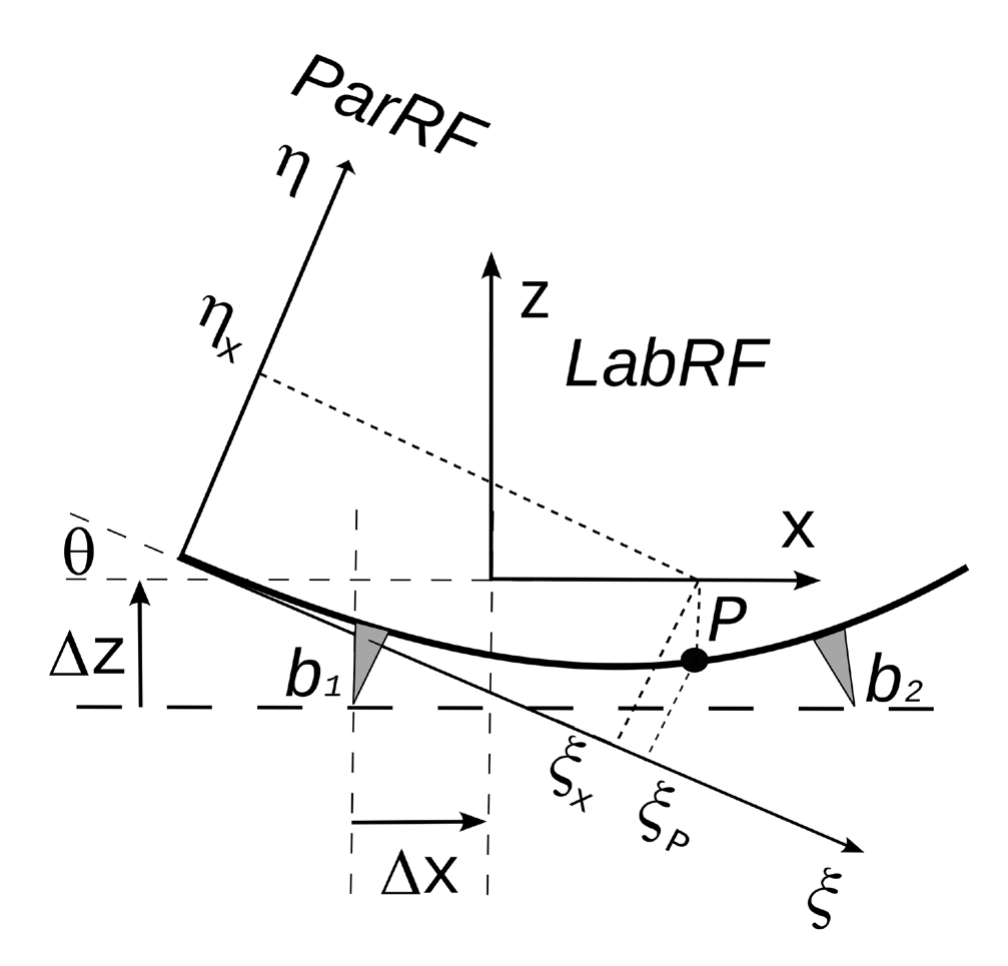
*ParRF* (parabola reference frame) and *LabRF* (lab reference frame); the center of the four supports (see Figure 1) lies over an horizontal plane parallel to the *X Y* plane of *LabRF*.

Referring to Figure 7, the angle *θ* between the *x* axis of *LabRF* and *ξ* axis of *ParRF* is given by

$$\theta =\arctan\left(\frac{\eta_{b_{2}}-\eta_{b_{1}}}{\xi_{b_{2}}-\xi_{b_{1}}}\right)\;(8)$$

where ( *ξ*_*b*_1__, *η*_*b*_1__) and ( *ξ*_*b*_2__, *η*_*b*_2__) are the coordinates in *ParRF* of the inner ( *b*_1_) and outer ( *b*_2_) supporting points.

Let Δ *x* and Δ *z* be the increments to reach the *LabRF* origin from the center of *b*_1_; in *ParRF* the origin of *LabRF* is at

$$\begin{array}{l} \xi_{L}=\xi_{b_{1}}+\text{Δ}x\;\cos\theta -\text{Δ}z\;\sin\theta \\ \eta_{L}=\eta_{b_{1}}+\text{Δ}x\;\sin\theta +\text{Δ}z\;\cos\theta . \end{array}\;(9)$$

Let ( *x*, *y*, *z*) be the coordinate of the measured point *P* of the panel surface. In order to compare *z* with the value expected for the ideal parabola, the value of *ξ _P_*, i.e. the abscissa of *P* in *ParRF*, should be known. Developing the reasoning in the plane *y* = 0, the point ( *x*, 0, 0) in *ParRF* has coordinates

$$\begin{array}{l} \xi_{x}=\xi_{L}+x\;\cos\theta \\ \eta_{x}=\eta_{L}+x\;\sin\theta . \end{array}\;(10)$$

The coordinates of *P* ( *ξ _P_* , *η _P_*) are the solution of the equation system

$$\begin{array}{l} \eta =\frac{1}{4f_{p}}\xi^{2}\\ \eta -\eta_{x}=-\frac{1}{\tan\theta }(\xi -\xi_{x}) \end{array}\;(11)$$

having solution

$$\xi_{P}=\frac{-b+\sqrt{b^{2}-4ac}}{2a}\;(12)$$

where $a=\frac{1}{4f_{p}}$ $b=\frac{1}{\tan\theta }$ $c=-\eta_{x}-\frac{\xi_{x}}{\tan\theta }$ .

Then, in *LabRF*

$$\begin{array}{l} x_{P}=(\xi_{P}-\xi_{L})\cos\theta +(\eta_{P}-\eta_{L})\sin\theta =x\\ z_{P}=-(\xi_{P}-\xi_{L})\sin\theta +(\eta_{P}-\eta_{L})\cos\theta \end{array}\;(13)$$

where $\eta_{P}=\frac{1}{4f_{p}}\xi_{P}^{2}$ .

Concerning the ideal slopes at point *P* in *LabRF*

$$\text{d}z/\text{d}x=\tan\left[\text{arctan}\left(\frac{\xi_{P}}{2f}\right)-\theta \right]\;(14)$$

dz/dy=0.(15)

Finally, the compliance of the measured 3D shape with the ideal one is given by the *deviation*, i.e. the difference between experimental and ideal values in *LabRF* has already discussed at the end of Section 2.7.

### 2.9 Intercept factor

The *Intercept Factor* (I-F) is an indicator of the actual efficiency of the panel when used to reflect the solar radiation onto the receiver; it is defined as the portion of the reflected radiation geometrically intercepted by the receiver. For PT panels, the intercept factor depends on the longitudinal *θ _L_* angle formed by the solar radiation with the collector axis: let us consider a single point on the panel surface; the solar radiation reflected at that point is shaped as a cone where the (half) apex angle is driven by the (half) divergence angle of the solar radiation on Earth *_S_*; the path length *t* between the point of reflection and the receiver tube depends on both the abscissa of the reflection point in *ParRF* and *θ _L_*; the radius of the spot-size of the reflected conic beam at the crossing of the receiver tube is *R* = *t _S_*. The software evaluates the intercept factor as the portion of the spot area which is geometrically intercepted by the receiver tube.

More precisely, the computing is run on each populated cell of the sampling matrix S: i) the experimentally determined components of the normal unit vector in *LabRF* are transformed in *ParRF*; ii) the unit vector of the specular reflection is evaluated on the basis of the unit vectors describing the impinging solar radiation (it depends on *θ _L_*) and the normal to the mirror surface; iii) the path length *t* between cell and receiver as well as the distance between the receiver axis and the specular ray (the axis of the reflected conic beam) are numerically computed; iv) finally the intercept factor is evaluated as the portion of the conic-beam spot intersecting the receiver tube.

The mean intercept factor is the arithmetic average of the local values at each of the populated cells of the sampling matrix S.

## 3 Validation experiment

VISproPT was designed for measuring Italian style PT panels; they are characterized by the use of 1 mm thick solar mirrors glued to rigid parabolic-shaped sheet moulding compound substrates, 1200 mm wide; so that a PT module (12 m long) is composed by 40 panels, 20 inner and 20 outer; the focal length is 1810 mm.

Conversely the panels adopted in the RR are for collector type LS3 with focal length 1710 ± 1 mm. The panel width is 1700 ± 1 mm while the chord is 1624 and 1501 mm for inner and outer type, respectively; that width value is a bit over the limit of the actual VISproPT hardware: although the two cameras can be placed up to 1.7 m apart (each one flying over one of the two curved-rims), the point source array is not sufficiently long to be viewed reflected over more than half-width of the panel surface when the cameras are close to *x _max_*; therefore a small triangular area with base centered in the middle of the farthest rim from the vertex is not sampled; in the 2D contour maps that not-sampled area is painted gray.

VISproPT was calibrated in the modified arrangement by minimizing the slope deviations of a perfectly flat surface as that of a calm body of water (see Section 2.6), keeping the parameters listed in Table 1 fixed to the value measured with the Total Station; here *x _cj_* and *z _cj_* are two of the three coordinates in *LabRF* of the lens center of the j-camera.

**Table 1.** Parameters measured by the Total Station. PSA=point source array. The error is 0.3 mm for coordinates and 0.1 deg for angles.

Parameter	Experimental value	unit
Center of PSA	(−760.0 , 0.0 , 1483.7)	mm
Yaw Pitch Roll of PSA	(0.0 ,−30.6 , 0.0)	deg
Δ *x*_21_ = *x*_*c*2_ − *x*_*c*1_	-3.2	mm
Δ *z*_21_ = *z*_*c*2_ − *z*_*c*1_	1.6	mm

In order to minimize the uncertainty, the full set of RR specimens (5 inner + 5 outer) was measured in the same session with the scan-parameters reported in Table 2.

**Table 2.** Main VISproPT parameters used to measure the round-robin specimens; *M* is cart position on the motorized rail.

Parameter	Inner	Outer
*M*_*max*_ (mm)	1960.0	2070.0
*M*_*min*_ (mm)	340.0	110.0
*M*_*step*_ (mm)	10.0	10.0
*M*4 *set*	1095.6	1095.6
Gray threshold	50	55

**Figure 8.**
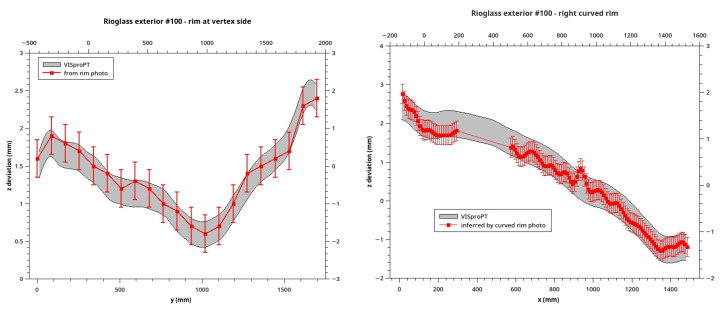
*z* deviation at two rims of Outer#100 obtained by VISproPT (grey band) and photograph (red error-bars).

The validation of the VISproPT results with those obtained with another independent technique is difficult. The most immediate solution might seem the Total Station, but unfortunately its Corner Cube Reflector accessory ^10^ cannot be placed freely on the surface of the panel because its weight-force deforms the panel surface itself, altering the result. Another approach, completely safe but limited, is the evaluation of the rim-shape by the analyses of its photo acquired from the side. Because of the VISproPT collocation in the Lab Hall, only two rims of the panel can be properly photographed. We use a Nikon D800 equipped with a Nikkor 24-120mmf3.5-5.6D. In each photo, the rim was manually sampled by reading the pixel-coordinates at several points, then transformed in millimeters by scaling them to the total length of the rim, or to the chord for the curved one. Finally the deviation from the ideal profile (linear for the edge close to the vertex and parabolic for the other) was calculated and compared with the ones obtained by VISproPT: as shown in Figure 8 the agreement is perfect although the VISproPT data-deviation refers to a section 10 mm far from the rim. Please note that, because of the shadowing of the panel by one of the four columns of the VISproPT structure, a small part of the curved rim is not visible, and deviation-data are missing.

**Figure 9.**
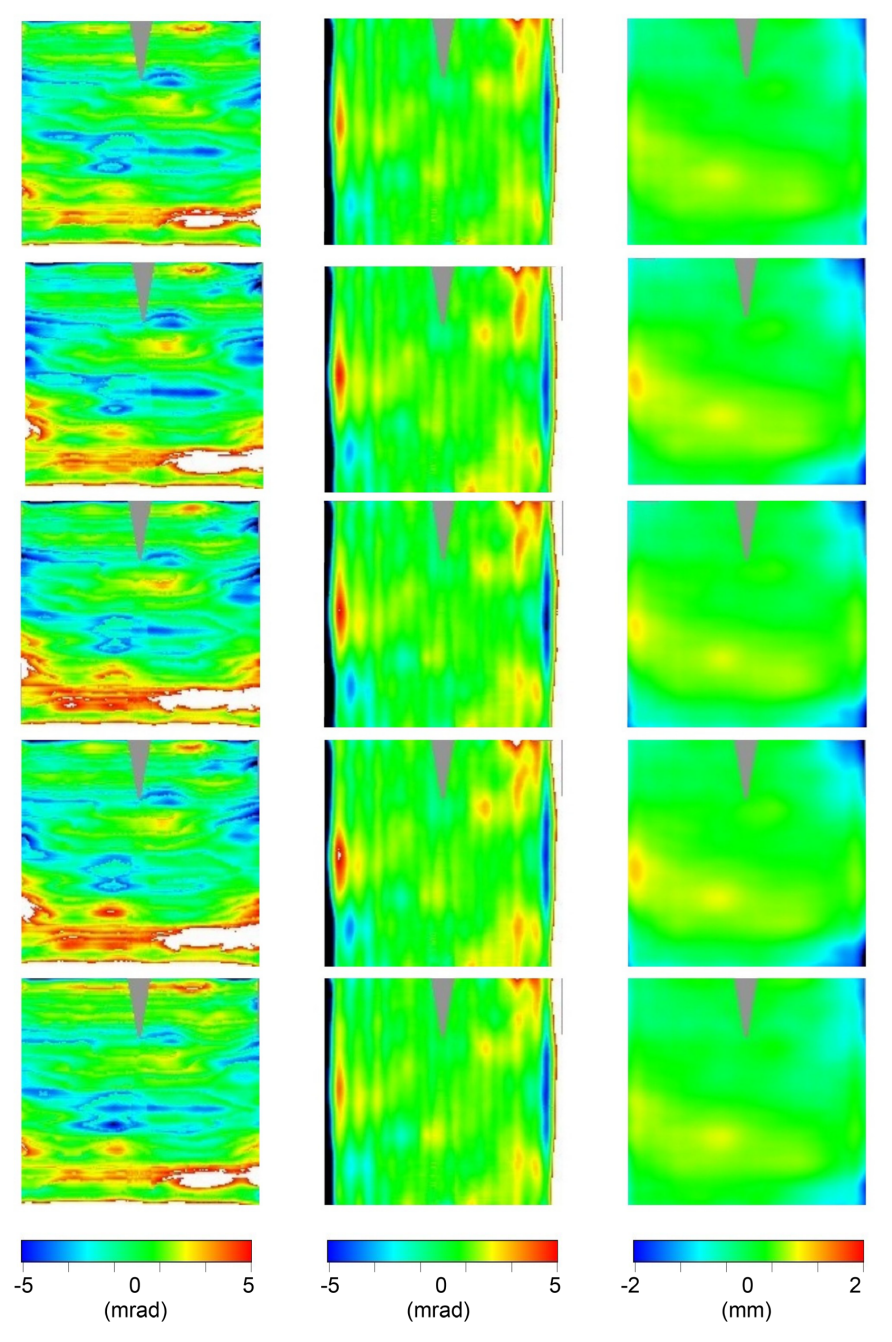
2D contour-maps of Δd *z/*d *x* (1st column), Δd *z/*d *y* (2nd column), and Δ *z* (3rd column) of the inner panels #58, #59, #60, #61, and #62 (sorted by rows). Color-range: [blue,red]= [-5,+5] mrad or [-2, 2] mm; black (white) below (above) that range; gray not sampled.

**Figure 10.**
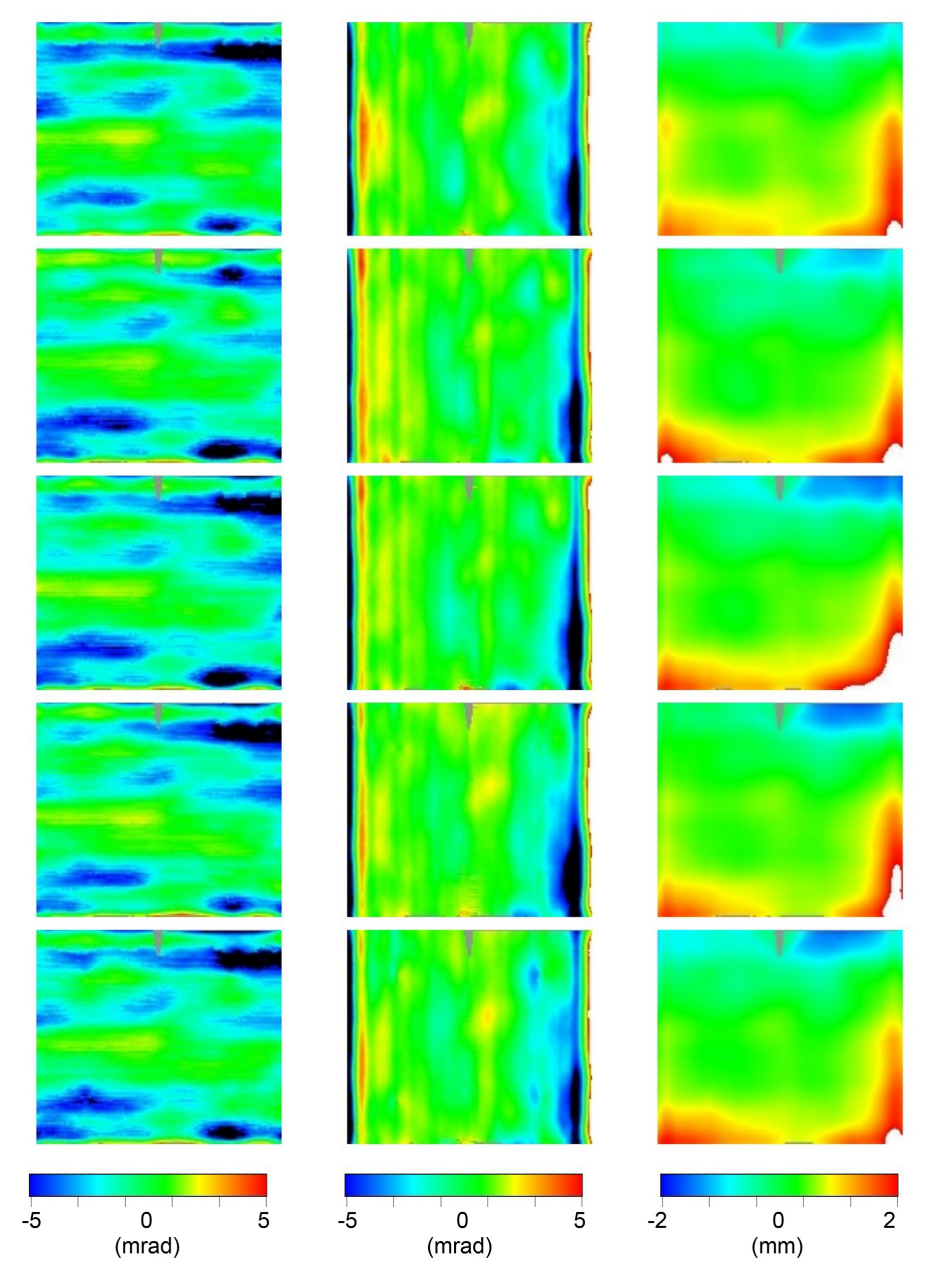
2D contour-maps of Δd *z/*d *x* (1st column), Δd *z/*d *y* (2nd column), and Δ *z* (3rd column) of the outer panels #93, #97,#99, #100, and #101 (sorted by rows). Color-range: [blue,red]= [-5,+5] mrad or [-2, 2] mm; black (white) below (above) that range; gray not sampled.

In order to show the instrument effectiveness, in the following the results we achieved on the RR specimen set (5 inner + 5 outer pannels) are reported. In particular, the results of the 3D shape measurement are here reported as deviation from the ideal values (see Section 2.8): Table 3 reports the RMS value of the deviation, while Figure 9 and Figure 10 show the 2D contour-maps, respectively for inner and outer panel. Both RMS values and 2D contour-maps are obtained by the multidimensional sampling matrix S[j][i][k] introduced in Section 2.7; more precisely, the contour maps are obtained by directly assigning at the pixel of the map with coordinates [j][i] the color from blue to red proportionally to the experimental value in the range [-5,+5] mrad and [-2, 2] mm for Δd *z/*d *x* or Δd *z/*d *y* and Δ *z*, respectively; in white (black) the value above (below) the considered range; in gray the not sampled area.

**Table 3.** Root mean square deviation from the ideal shape.

Panel	Δd *z*/d *x* (mrad)	Δd *z*/d *y* (mrad)	Δ *z* (mm)
Inner#58	2.1	2.7	0.40
Inner#59	2.5	2.6	0.50
Inner#60	2.6	2.7	0.47
Inner#61	2.6	2.7	0.49
Inner#62	2.1	2.7	0.40
Outer#093	1.6	2.1	0.71
Outer#097	1.6	2.0	0.68
Outer#099	1.6	2.1	0.79
Outer#100	1.6	2.3	0.70
Outer#101	1.5	2.1	0.73

Panels belonging to the same kind (inner and outer) resulted to be affected by very similar deviations; probably this is a consequence of the good systematic nature of the production process which is affected by some imperfections.

Once the 3D shape of the panel is known, the intercept factor can be evaluated. The computation is made by considering the standard value of the solar radiation divergence, 4.7 mrad (half-apex angle), and three different values for the longitudinal incidence angle ( *θ _L_*): 0, 35 and 70 deg.

**Figure 11.**
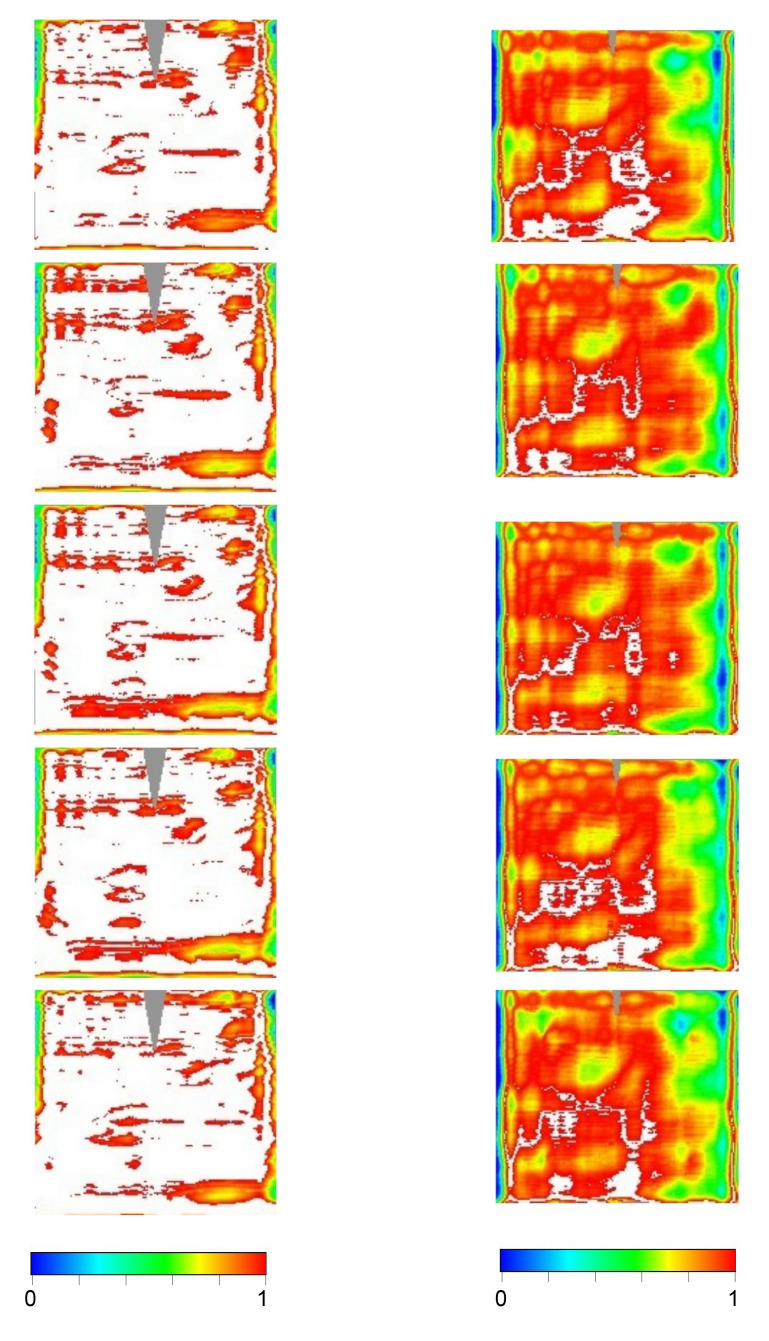
2D contour-maps of intercept factor at *θ _L_* = 70° for inner (left) and outer (right) panels, sorted by rows. Color-range: [blue,red] = [0,1); white full interception; gray not sampled.

Table 4 summarizes the intercept-factor mean-values, while Figure 11 shows the intercept factor as 2D contour-map at 70 deg; here the white color is assigned for full interception, while colors from blue to red are used to indicate values from 0 to 1.

from 0 to 1.

**Table 4.** Mean intercept factor.

Panel	*θ _L_* = 0°	*θ _L_* = 35°	*θ _L_* = 70°
Inner#58	1.000	1.000	0.976
Inner#59	1.000	0.999	0.969
Inner#60	0.999	0.999	0.966
Inner#61	0.999	0.998	0.966
Inner#62	1.000	1.000	0.973
Outer#093	0.993	0.982	0.826
Outer#097	0.998	0.994	0.844
Outer#099	0.993	0.987	0.825
Outer#100	0.992	0.985	0.824
Outer#101	0.993	0.983	0.815

As expected the intercept factor mainly depends on the Δd *z/*d *x* deviation, but for the outer panels, at oblique incidence, also the Δd *z/*d *y* deviation reduces the intercept factor. Although the shape of the outer panels is closer to the ideal parabola than the inner ones (see Table 3), in terms of intercept factor, the outer panels perform less well because of the longer optical path between point of incidence and receiver tube; the effect becomes more evident as the longitudinal angle *θ _L_* increases.

## 4 Conclusions

The VISproPT instrument is the engineered version of the older VISprofile ^6^. The hardware was manufactured by MARPOSS Italia Spa ^7^ and funded by the SFERA-III EU project ^8^, while ENEA developed the image processing software as well as the calibration procedure.

Differently from VISproLF ^5^, the instrument is not completely self-calibrating because some components have to be aligned and positioned with high accuracy; to that aim we used a Total Station.

The instrument is designed for indoor measuring the 3D shape of PT panels, but the outlined image processing method is also effective for differently curved specimens. As an example, in the current configuration, VISproPT can also measure flat specimens, like those for linear Fresnel plants, as long as their length does not exceed 1 m; however this limit could be easily overcome by improving the hardware to accomplish the scan by moving the specimen, while keeping the point source array and the cameras in steady position.

The fine-calibration of the instrument is based on the shape measurement of a calm body of water. After that, the instrument accuracy resulted to be better than 0.1 mrad and 0.3 mm (RMS value over an area 1.2×0.8 m ^2^), respectively for slope and height of the surface. In a following paper dealing with the results of the round-robin, the accuracy of all the adopted instruments will be compared and discussed.

The VISproPT was exploited by measuring ten panels (5 inner + 5 outer) used in the round-robin (RR) on 3D shape measurements organized in the framework of the SFERA-III EU project, with the participation of F-ISE, DLR, NREL and SANDIA. The scansion of a specimen and the following image-processing typically take less than 90 s by a PC with Intel Xeon CPU E5-1620 v4 @ 3.50GHz; the duration of the measurement would be shorter using a more modern and performing PC.

For the sake of RR reliability, ENEA developed a simple supporting system which was proven to ensure a satisfactory repeatability of the specimen-placing; supporting system and instructions will circulate together with the panels.

The deviation from the ideal parabolic profile are quite similar among the panels belonging to the same type, inner and outer; typically the slope deviation is better than 3 mrad and 2 mrad, respectively for inner and outer. The intercept factor at *θ _L_* = 0° is better than 99.9% and 99.2%, respectively for inner and outer kind.

## Ethics and consent

Ethical approval and consent were not required.

## Data Availability

Zenodo: mmonty1960/VISproPT: v2 http://doi.org/10.5281/zenodo.7889928 ^15^. This project contains the following underlying data as part of the software repository: RGinterior#58.7z (7zip-file containing the sequences of images acquired by the two cameras of the VISproPT for the exemplary inner PT panel #058). AcamNew.txt (setting-file with the instrument parameters used for measuring all the inner panels). Data are available under the terms of the GNU General Public License version 3.

## Software availability

Source code available from: https://github.com/mmonty1960/VISproPT

Archived source code at time of publication: http://doi.org/10.5281/zenodo.7889928 ^15^

License: GPL-3.0-only

## References

[1] Montecchi M : Italian Patent. RM2008A000151,2008.

[2] Montecchi M Benedetti A Cara G : Optical alignment of parabolic trough modules. 2010. Reference Source

[3] Montecchi M Casa MD : Post-assembly in-situ check of parabolic trough modules by VISshed. *AIP Conf Proc.* 2018;2033: 030009. 10.1063/1.5067025

[4] Montecchi M Cara G Benedetti A : Visdish: A new tool for canting and shape-measuring solar-dish facets. *Rev Sci Instrum.* 2017;88(6): 065107. 10.1063/1.4984944 28667978

[5] Montecchi M Cara G Benedetti A : Visprolf: Self-calibrating instrument for measuring 3d shape of linear fresnel facets. *Rev Sci Instrum.* 2020;91(8): 083109. 10.1063/5.0013116 32872943

[6] Montecchi M Benedetti A Cara G : Fast 3d optical-profilometer for the shape-accuracy control of parabolic trough facets.september, 2011. Reference Source

[7] Marposs italia spa. 2023. Reference Source

[8] Sfera-iii, solar facilities for the european research area. 2023. Reference Source

[9] Solarpaces task iii: Solar technology and advanced applications. 2022. Reference Source

[10] Leica total station tda5005. 2022. Reference Source

[11] Qt: cross-platform application development framework for desktop, embedded and mobile. 2019. Reference Source

[12] Open source computer vision library. 2019. Reference Source

[13] Devernay F : C/c++ minpack. 2017. Reference Source

[14] Montecchi M : Vispropt. 2022. Reference Source

[15] mmonty1960: mmonty1960/VISproPT: v2 (Version V2). *Zenodo* . [software],2023. 10.5281/zenodo.7889928

[16] Euler angles. 2020. Reference Source

[17] Ydrissi ME Ghennioui H Bennouna EG : A review of optical errors and available applications of deflectometry technique in solar thermal power applications. *Renew Sustain Energy Rev.* 2019;116: 109438. 10.1016/j.rser.2019.109438

