# Supplementary material for: VISproPT: A high-precision instrument for 3D shape analysis of parabolic trough panels
Source: Open Res Eur. 2023 Nov 28;3:111. Originally published 2023 Jul 10. [Version 2] doi: 10.12688/openreseurope.15967.2 (PMC10511855; doi:10.12688/openreseurope.15967.2)
Supplement: Peer review history

# Reviewer report: Reviewer response for version 2

**DOI:** 10.21956/openreseurope.18235.r36479

**Published:** 2023-12-04

**Authors:** İbrahim Halil Yılmaz

**Affiliations:** 1 Department of Mechanical Engineering, Adana Alparslan Turkes Science & Technology University, Adana, Turkey

The authors have addressed the comments with reasonable responses. The conceptualization and technical synthesis of the manuscript look better in its current form.

Is the rationale for developing the new method (or application) clearly explained?

Yes

Is the description of the method technically sound?

Yes

Are the conclusions about the method and its performance adequately supported by the findings presented in the article?

Yes

If any results are presented, are all the source data underlying the results available to ensure full reproducibility?

Yes

Are sufficient details provided to allow replication of the method development and its use by others?

Yes

Reviewer Expertise:

Concentrating solar thermal, parabolic trough collectors, heat transfer enhancement

I confirm that I have read this submission and believe that I have an appropriate level of expertise to confirm that it is of an acceptable scientific standard.

# Reviewer report: Reviewer response for version 1

**DOI:** 10.21956/openreseurope.17241.r34807

**Published:** 2023-09-20

**Authors:** Qiliang Wang

**Affiliations:** 1 Department of Building Environment and Energy Engineering, The Hong Kong Polytechnic University, Hong Kong, Hong Kong

This study presents a novel instrument called VISproPT, which boasts high precision, to validate the shape compliance of parabolic trough panels. It is an excellent contribution that holds great potential for the advancement of solar thermal power. I have a couple of minor questions that I would appreciate your further consideration on:

1. To enhance clarity, it would be beneficial to provide a more detailed explanation of the color maps depicted in Figures 9 and 10, highlighting the differences and advancements they demonstrate.
2. It would be helpful to include a comparison of the economic performance of the instrument with other similar instruments, to further illustrate its advantages.

Is the rationale for developing the new method (or application) clearly explained?

Yes

Is the description of the method technically sound?

Yes

Are the conclusions about the method and its performance adequately supported by the findings presented in the article?

Yes

If any results are presented, are all the source data underlying the results available to ensure full reproducibility?

Yes

Are sufficient details provided to allow replication of the method development and its use by others?

Yes

Reviewer Expertise:

Concentrated Solar Thermal, Solar Thermal Utilization

I confirm that I have read this submission and believe that I have an appropriate level of expertise to confirm that it is of an acceptable scientific standard.

# Response

**Published:** 2023-09-21

**Authors:** Marco Montecchi

**Affiliations:** ENEA Casaccia Research Center, Italy

We thank the referee for his comments. Concerning the first, Fig. 9 and 10 are shown just to demonstrate the instrument working; they do not in themselves prove any advancement. Considering also the second comment, the referee's underlying main suggestion becomes clear: the comparison with the other similar instruments. Of course we completely agree with the importance of the point raised by the referee. On the other hand, as declared, the present paper deals with only the VISproPT instrument. The results of the round-robin will be presented in a next paper as well as a critical comparison of advantages and disadvantages of the different approaches and instruments.

# Reviewer report: Reviewer response for version 1

**DOI:** 10.21956/openreseurope.17241.r34805

**Published:** 2023-09-11

**Authors:** İbrahim Halil Yılmaz

**Affiliations:** 1 Department of Mechanical Engineering, Adana Alparslan Turkes Science & Technology University, Adana, Turkey

The article proposes a visual inspection approach based on the idea of placing a light source near the focus of the reflector and several images taken in the near-field from different positions of the cameras used. The method followed is well-presented and clearly described nonetheless there are a few unclear points that should be technically recompiled for the conceptualization of the study and interested audience. I recommend some comments listed below that would enhance the technical view of the article and its clarity:

1. Spelling, punctuation, typos, and missing spaces should be corrected. The text needs thorough proofreading once again for clarity and scientific accuracy. In addition, abbreviations must be written explicitly where they are first used without continuously repeating them. Please check the main text and edit them all.
2. Section 2.4 and 2.5: The home position for CamRF, i.e. (0, 0, 0), should be specified clearly in the main text as the center of the chessboard. It is shown in Fig. 3 as the principle point but Eq. (5) is applicable under the mentioned circumstance for corners.
3. There are previously defined but later explained symbols or parameters such as yaw, pitch, and roll which need to be ordered for fluency. Please check, if any.
4. Section 2.6: It could be giving more detail about how the instrument optimization was adapted for cameras by the modified Levenberg Marquardt nonlinear least square algorithm.
5. It would be better to mention the instrument accuracies describing the total system calibration and their sensitivities to laboratory conditions. Any factor that would be effective throughout the procedure would be discussed. Any tolerance or statistical data could be given for clarity.
6. Eq. (14): Please use × symbol for the multiplication process.
7. A simple benchmark table could be added for comparing round-robin tests.
8. It could be better to present the computation of the intercept factor.
9. Table 4: Please discuss why the intercept factors of the outer panels at 70° are lower than those of the inner ones.
10. Figs. 9, 10 and 11: The color range could be shown by a heat map scale.
11. Ref. 17: "appli- cations" need to be edited.

Is the rationale for developing the new method (or application) clearly explained?

Yes

Is the description of the method technically sound?

Yes

Are the conclusions about the method and its performance adequately supported by the findings presented in the article?

Yes

If any results are presented, are all the source data underlying the results available to ensure full reproducibility?

Yes

Are sufficient details provided to allow replication of the method development and its use by others?

Yes

Reviewer Expertise:

Concentrating solar thermal, parabolic trough collectors, heat transfer enhancement

I confirm that I have read this submission and believe that I have an appropriate level of expertise to confirm that it is of an acceptable scientific standard.

# Response

**Published:** 2023-09-14

**Authors:** Marco Montecchi

**Affiliations:** ENEA Casaccia Research Center, Italy

Many thanks to the referee for his comments and questions that will be considered in drafting the next paper version. In the meanwhile, our answers are following.

1. The next version of the paper will be carefully revised; the acronym explanation will be always given at its first appearance.
2. The data processing involves 5 different reference frames, making the full comprehension of the proposed work-flow a bit difficult. The question set by the referee makes us understand that we need to improve the explanation. In particular, CamRF1 is the reference frame related to Camera#1; similarly CamRF2 is the reference frame of Camera#2. As explained in Section 2.3 the origin of these references is set on the “lens center” of the system camera+lens; the “principal point” is the orthogonal projection of the lens center to the sensor plane (generally it differs from the center of the sensor); the coordinates of the principal point in CamRF are determined (together the distortion coefficients) by means of the camera calibration procedure. The center of the chessboard shown in Fig. 4 is the origin of the Laboratory reference frame (LabRF). Before getting the chessboard images displayed in Fig.4, the camera position along the motorized rail is adjusted to “x4set”, that is the current value to observe the center of the image acquired by Camera#1 superimposed to the line x=0 (in LabRF) of the chessboard. After that the coordinates in LabRF of the lens center (the origin of CamRF) as well as its attitude (Yaw, Pitch, Roll) are evaluated by best fitting the expected corner position in the image of the chessboard, to the experimental values obtained by the image, as explained in Section 2.5; this process is run independently for each camera.
3. In the next version we will carefully check the punctual explanation of each term/symbol at its first appearance.
4. The instrument final calibration will be more clearly explained.
5. The Leica total station TDA5005 is a well assessed commercial instrument; the information required by the referee are given in reference [10]; in this work we consider the TDA5005 as a proved benchmark instrument. Concerning the laboratory condition, several heat pumps work h24, maintaining the temperature to 23 °C during the whole year; the humidity is not explicitly controlled, even if the cooling system avoids the occurrence of excess. Along the instrument development, total station measurements have been repeated in different periods of the year, but we never observed deviation greater than the declared experimental error of the TDA5005 (0.3 mm).
6. The symbol “*” will be omitted because unnecessary; it is a residue of the copy and past of the C++ code.
7. The results of the round-robin will be reported in a separated paper.
8. The intercept computing will be described in the next version of the paper.
9. The path-length between the point of reflection and the receiver increases with the abscissa (in the parabola reference frame ParRF) of the reflection point; this length increases with the incidence angle. That makes the performances of the outer panels at 70°C worst.
10. A legend of the colour scale will be added to figures 9, 10 and 11.
11. The mistyping in ref [17] will be corrected.

# Reviewer report: Reviewer response for version 1

**DOI:** 10.21956/openreseurope.17241.r33630

**Published:** 2023-07-28

**Authors:** Jesus Fernandez-Reche

**Affiliations:** 1 CIEMAT–Plataforma Solar de Almería, Tabernas-Almería, Spain

I congratulate the authors for the article, its technical level and completeness; but above all, for making this methodology available to the CSP community, since the complete description included in the article, together with the software developed and the experimental data used in it, makes this methodology easily replicable.

 I would just like to add a couple of comments that, I hope, will improve the already good technical quality of the article.

1. In the introduction of the article, when talking about the intercomparison campaign carried out under the SFERA III project, 10 reflector specimens are mentioned, but it is not specified whether they are PT, Heliostat, Fresnel, etc. The context would be better understood if the type of reflectors were explicitly indicated.
2. Section 2.1, where the basis of the methodology is described, should indicate what kind of environmental conditions the methodology requires. As this is a high-precision system assembled in a metallic structure, I understand that a temperature, humidity, and/or movement control is necessary to ensure the accuracy and replicability of the measurements. Please add this in the description of the system, as well as the accuracies obtained in the positioning of the main components (cameras, LED array, measurement plane) and the pressures on the individual LEDs that make up the measurement matrix.
3. Although the colour range is included in the text, a legend of the colour scale used in figures 9, 10 and 11 would improve the interpretation of the figures.
4. The proposed methodology calculates the normal vectors to the surface of the reflectors at those locations where there is a reflected image from the LEDs included in the system. Therefore, in figures 9, 10 and 11 a spatial interpolation has been performed between these positions to extend the results to the whole surface. Could the authors please indicate what kind of interpolation has been performed?
5. Have the overall results from tables 3 and 4 been calculated only from the experimental data, or has data from such spatial interpolation been included in the calculation of the results?

Congratulations again for the article.

Is the rationale for developing the new method (or application) clearly explained?

Yes

Is the description of the method technically sound?

Yes

Are the conclusions about the method and its performance adequately supported by the findings presented in the article?

Yes

If any results are presented, are all the source data underlying the results available to ensure full reproducibility?

Yes

Are sufficient details provided to allow replication of the method development and its use by others?

Yes

Reviewer Expertise:

Solar Energy, CSP, 3D geometrical characterization

I confirm that I have read this submission and believe that I have an appropriate level of expertise to confirm that it is of an acceptable scientific standard, however I have significant reservations, as outlined above.

# Response

**Published:** 2023-08-03

**Authors:** Marco Montecchi

**Affiliations:** ENEA Casaccia Research Center, Italy

First of all we would like to thank the referee for his comments and questions that will drive the  drafting of the next paper version. In the mean while, our answers are following.

1. The type of reflector (PT) will be added in the abstract.
2. The Lab is equipped with several heat pumps working h24; they are set to maintain the temperature to 23 °C along the year; the humidity is not explicitly controlled, even if the cooling system avoid the occurrence of excess. Along the instrument development, total station measurements have been repeated in different period of the year, but we never observed deviation greater than its experimental error (0.3 mm). In any case, after any set-up modification, the final calibration over a calm body of water must be run, thus many parameters of the VISproPT are newly optimized.
3. A legend of the colour scale will be added to figures 9, 10 and 11.
4. All the surface parameters (normal vector, slopes and z) are evaluated by combining blob (image) and its corresponding point source; they are stored in the cell of the sampling matrix crossed by the vertical line originating from the given reflection point. With the instrument setting reported in the paper, generally the same cell of the sampling matrix is populated with about 5 different independent evaluations; then the arithmetic mean value of each parameters is considered and definitively associated to the cell. The great advantage of the sampling matrix is the regularity of the data density and distribution across the plane XY of the LabRF.
5. The results of Tab. 3 and 4 are computed starting from the values stored in the sampling matrix.
